# Supplementary material for: Lifestyle patterns and their nutritional, socio-demographic and psychological determinants in a community-based study: A mixed approach of latent class and factor analyses
Source: PLoS One. 2020 Jul 23;15(7):e0236242. doi: 10.1371/journal.pone.0236242 (PMC7377498; doi:10.1371/journal.pone.0236242)
Supplement: S1 File — (PDF) [file pone.0236242.s002.pdf]

دانشگاه علوم پزشکی
و خدمات بهداشتی درمانی تبریز

Tabriz University of Medical Sciences

Food Frequency Questionnaire (FFQ)

Patient's name and surname:

Items	Foods	Amounts	Day	Week	Month	Year	Additional explanations
1	Lavash bread						
2	Rice						
3	French bread						
4	Other breads						
5	Pasta						
6	Noodles						
7	Barbari bread						
8	Sangak bread						
9	Jo bread						
10	Brown or wholegrain breads						
11	Potato						
12	Milk						
13	Chocolate milk, coffee milk, flavoured milk						
14	Yogurt						
15	Cheese						
16	Kashk						
17	Doogh (yogurt drink)						
18	Icecream						
19	dairy based						
20	Red meat						
21	Chicken, turkey, duck						
22	eggs						
23	Fish						
24	Citrus						
25	Apple						
26	Dates						
27	Dried fruits						
28	Banana						
29	Peaches or nectarines						
30	Kiwifruit						
31	Strawberries or berries						
32	Grapes						
33	Melons (including watermelon, rockmelon)						
34	Pineapple						
35	Other fruits						

36	Fruit juices						
37	Pears						
38	Apricots						
39	Plums						
40	Tomatoes (cooked or raw)						
41	Cucumber						
42	Lettuce (salad/leaves)						
43	Cabbage (white/red)						
44	Cauliflower, Broccoli, Brussels sprout						
45	Green leafy vegetables						
46	raw as side dish						
47	Green leafy vegetables						
48	cooked in dish						
50	Spinach (raw/cooked)						
51	Onion (raw/cooked)						
52	Roots vegetables						
53	Mushrooms						
54	Corn						
55	Taro						
56	Green beans, mung pea, lentil						
57	Soy protein						
58	pea						
59	bean						
60	Walnuts						
61	Almonds						
62	Peanuts						
63	Pistachios & hazelnuts						
64	Raisons						
65	dried peas						
65	Seeds (sunflower, watermelon, pumpkin)						
66	Non- hydrogenated vegetable oil, olive oil						
66	Soft margarine						
67	Ghee, butter, cream						
68	Visceral fat and liver, Kidney, Heart and other organ meats						
69	Cookies/biscuits, Cakes, Pastries						

70	Chocolate/bars, Sweet, Cookie, jam						
71	Coke						
72	Diet coke						
73	Canned fruits and industrial fruit juices						
74	Frankfurter						
75	Sausages						
76	Hamburger						
77	Pizza						
78	Canned foods						
79	Sour and salty pickles						
80	Tea or coffee						
