# Supplementary material for: Lifestyle patterns and their nutritional, socio-demographic and psychological determinants in a community-based study: A mixed approach of latent class and factor analyses
Source: PLoS One. 2020 Jul 23;15(7):e0236242. doi: 10.1371/journal.pone.0236242 (PMC7377498; doi:10.1371/journal.pone.0236242)
Supplement: S2 File — (PDF) [file pone.0236242.s003.pdf]

پرسشنامه بسامد مصرف خوراک

ردیف	مواد غذایی	مقدار	متوسط *بار مصرف* در سال گذشته				میزان مصرف در *هر بار*	ماه در سال	ملاحظات
			روز	هفته	ماه	سال			
F1	نان و غلات								
۱	نان لواش	کف دست/ کامل							
۲	نان بربری/تافتون	کف دست/ کامل							
۳	نان سنگک	کف دست/ کامل							
۴	نان باگت و فانتزی	یک عدد							
۵	سایر نان ها	کف دست /کامل							۱.نان سنتی<br>۲.نان روغنی
۶	برنج پخته	کفگیر / بشقاب							۱. سفید<br>۲. سبوس دار
۷	ماکارونی پخته	کفگیر / بشقاب							
۸	جو پخته /بلغور	قاشق غذاخوری							
F2	حبوبات								
۹	عدس	قاشق غذاخوری							
۱۰	لوبیاسفید،چشم بلبلی،قرمز، چیتی	قاشق غذاخوری							
۱۱	لپه	قاشق غذاخوری							
۱۲	نخود	قاشق غذاخوری							
۱۳	باقلای پخته (پلو/ زمستانی)	قاشق غذاخوری							
۱۴	سویا (پروتئین سویا ، دانه سویا)	قاشق غذاخوری							
F3	گوشت و فرآورده های آن								
۱۵	گوشت قرمز<br>(آبگوشتی، خورشتی، چرخ کرده)	قوطی کبریت							۱-کم چرب<br>۲-پرچرب
۱۶	گوشت مرغ	قوطی کبریت							۱-با پوست<br>۲-بدون پوست
۱۷	تخم مرغ	عدد							
۱۸	ماهی	قطعه متوسط							
۱۹	تن ماهی	قاشق غذاخوری							
۲۰	سوسیس، کالباس، همبرگر	یک واحد							
۲۱	احشا: دل ، جگر و قلوه<br>(گوسفند،گوساله و..) سایر قسمتهای<br>مرغ (جگر، دل، سنگدان)	قوطی کبریت							
۲۲	کله، پاچه /سیرابی /شیردان/مغز/<br>زبان	بشقاب<br>غذاخوری							

شهرستان		کد ناحیه		کد خوشه			شماره خانوار		نام سرپرست		نام فرد پاسخگو	

ردیف	مواد غذایی	مقدار	متوسط *بار مصرف* در سال گذشته				میزان مصرف در *هر بار*	ماه در سال	ملاحظات
			روز	هفته	ماه	سال			
۲۳	**پیتزا**	برش							
**F4**	**شیر و لبنیات**								
۲۴	**شیر**	لیوان							۱. کم چرب<br>۲. پرچرب/ محلی<br>۳. شیر کاکائو
۲۵	**ماست**	لیوان							۱. کم چرب<br>۲. پرچرب/ محلی<br>۳. خامه ای<br>۴. چکیده
۲۶	**دوغ**	لیوان							
۲۷	**پنیر**	قوطی کبریت							۱. معمولی<br>۲. خامه ای<br>۳. محلی
۲۸	**کشک**	قاشق غذاخوری							
۲۹	**پنیر پیتزا**	قاشق غذاخوری							
**F5**	**سبزیجات**								
۳۰	**سالاد با کاهو یا کلم**	لیوان /پیش دستی							
۳۱	**خیار**	عدد متوسط							
۳۲	**گوجه فرنگی**	عدد متوسط							
۳۳	**هویج**	عدد متوسط							
۳۴	**سبزی خوردن**	لیوان /پیش دستی							
۳۵	**سبزی غذا (خورشتی/آش/ کوکو/دلمه)**	پیش دستی / لیوان							
۳۶	**کدو / بادمجان**	عدد متوسط							
۳۷	**کرفس خام یا پخته / کنگر/لوبیا سبز/ نخود سبز**	قاشق غذاخوری							
۳۸	**سیر**	حبه							
۳۹	**پیاز**	عدد متوسط							
۴۰	**چغندر (لبو)/ شلغم**	عدد متوسط							
۴۱	**فلفل دلمه ای/ فلفل سبز**	لیوان/پیش دستی							
۴۲	**ذرت و بلال**	عدد/ لیوان							
۴۳	**سیب زمینی آب پز یا بخارپز**	عدد متوسط							
۴۴	**سیب زمینی سرخ کرده**	عدد متوسط							
**F6**	**میوه جات**								

ردیف	مواد غذایی	مقدار	متوسط *بار مصرف* در سال گذشته				میزان مصرف در *هر بار*	ماه در سال	ملاحظات
			روز	هفته	ماه	سال			
۴۵	زردآلو/هلو/شلیل/شفتالو/آلو	عدد							
۴۶	گلابی /انار/ خرمالو	عدد							
۴۷	سیب/کیوی/موز	عدد							
۴۸	مرکبات(پرتقال،نارنگی،لیموشیرین،گریپ فروت)	عدد							
۴۹	گیلاس / آلبالو/توت	پیش دستی/پیاله							
۵۰	توت فرنگی/ گوجه سبز	عدد							
۵۱	خربزه/ هندوانه/طالبی	قاچ متوسط							
۵۲	انگور	خوشه متوسط							
۵۳	آبمیوه طبیعی	لیوان							
۵۴	آبمیوه صنعتی (ساندیس، رانی و سایر)	لیوان							
۵۵	خرما /انجیر خشک/ برگه هلو/ برگه آلو/برگه زردآلو	عدد							
۵۶	خشکبار (کشمش ، توت خشک)	قاشق غذاخوری							
F7	انواع روغن، دانه های روغنی و کره								
۵۷	روغن جامد/نیمه جامد	قاشق غذاخوری							۱. حیوانی<br>۲. گیاهی<br>۳. دنبه
۵۸	روغن مایع	قاشق غذاخوری							۱. کانولا/سویا<br>۲. سایر روغنهای مایع (آفتابگردان/ذرت)
۵۹	کره/خامه / سرشیر	قاشق مربا خوری							
۶۰	روغن زیتون/زیتون	قاشق غذاخوری/عدد							
۶۱	سس مایونز یا سس گوجه فرنگی	قاشق غذاخوری							
۶۲	بادام/گردو/پسته/فندوق/بادام زمینی	عدد							
۶۳	مغز تخمه کدو، آفتابگردان، هندوانه	قاشق غذاخوری							
F8	قند ها								
۶۴	قند	حبه							
۶۵	شکر	قاشق چای خوری							
۶۶	نبات/آبنبات/نقل/ شکرپنیر	حبه							
۶۷	شکلات صبحانه/عسل/مربا	قاشق مرباخوری							

ردیف	مواد غذایی	مقدار	متوسط *بار مصرف* در سال گذشته				میزان مصرف در *هر بار*	ماه در سال	ملاحظات
			روز	هفته	ماه	سال			
۶۸	**دوشاب**	قاشق غذاخوری							
**F9**	**متفرقه**								
۶۹	**آب**	لیوان							
۷۰	**چای/قهوه/نسکافه**	لیوان							
۷۱	**نوشابه و ماءالشعیر**	لیوان							
۷۲	**بستنی (سنتی /غیر سنتی/کیم)**	لیوان/عدد							
۷۳	**شیرینی خشک/بیسکویت /کیک/کلوچه/**	واحد							
۷۴	**شیرینی خامه دار/کیک خامه دار**	عدد/برش							
۷۵	**انواع چیپس و پفک و ذرت بوداده**	بسته متوسط							
۷۶	**شکلات**	عدد							
۷۷	**انواع حلوا (شکری، کنجدخانگی)**	قاشق غذاخوری							
۷۸	**خیارشور**	عدد/پیاله							
۷۹	**ترشی**	عدد/ پیاله							
**F10**	**ادویه جات**								
۸۰	**نمک**	قاشق چای خوری							یددار ☐ غیر یددار ☐
