# Supplementary material for: Lifestyle patterns and their nutritional, socio-demographic and psychological determinants in a community-based study: A mixed approach of latent class and factor analyses
Source: PLoS One. 2020 Jul 23;15(7):e0236242. doi: 10.1371/journal.pone.0236242 (PMC7377498; doi:10.1371/journal.pone.0236242)
Supplement: S3 File — (PDF) [file pone.0236242.s004.pdf]

دانشگاه علوم پزشکی
و خدمات بهداشتی درمانی تبریز

Tabriz University of Medical Sciences

Generalized Anxiety Disorder 7-item (GAD-7) scale

Over the last 2 weeks, how often have you been bothered by the following problems?	Not at all sure	Several days	Over half the days	Nearly every day
1.Feeling nervous, anxious, or on edge	0	1	2	3
2.Not being able to stop or control worrying	0	1	2	3
3.Worrying too much about different things	0	1	2	3
4.Trouble relaxing	0	1	2	3
5.Being so restless that it's hard to sit still	0	1	2	3
6. Becoming easily annoyed or irritable	0	1	2	3
7. Feeling afraid as if something awful might happen	0	1	2	3
Add the score for each column	+	+	+	+
Total Score (*add your column scores*)=				
