# Supplementary material for: Lifestyle patterns and their nutritional, socio-demographic and psychological determinants in a community-based study: A mixed approach of latent class and factor analyses
Source: PLoS One. 2020 Jul 23;15(7):e0236242. doi: 10.1371/journal.pone.0236242 (PMC7377498; doi:10.1371/journal.pone.0236242)
Supplement: S4 File — (PDF) [file pone.0236242.s005.pdf]

بررسی اثر بخشی برنامه بهبود شیوه زندگی

پرسشنامه اضطراب GAD-7 (۵)

	در طول ۲ هفته گذشته، هر چند وقت یکبار مسائل زیر باعث ناراحتی شما بوده است؟	هیچ وقت	هر چند روز	بیشتر روزها	تقریبا هر روز
۱.	احساس عصبانیت و اضطراب	۰	۱	۲	۳
۲.	عدم توانایی در توقف یا کنترل نگرانی یا دلواپسی	۰	۱	۲	۳
۳.	نگرانی بیش از حد در مورد چیزهای مختلف	۰	۱	۲	۳
٤.	عدم توانایی در حفظ آرامش	۰	۱	۲	۳
۵.	به اندازه‌ای بیقرار باشید که حتی امکان نشستن هم نداشته باشید	۰	۱	۲	۳
٦.	به راحتی خشمگین یا تحریک پذیر شدن	۰	۱	۲	۳
۷.	احساس نگرانی از احتمال وقوع یک اتفاق ناگوار	۰	۱	۲	۳

پرسشنامه ایمنی غذایی (٦)

Q1- به نظر شما جگر نیمه پخته منجر به ایجاد مسمومیت غذایی می گردد؟ ۱) بلی☐ ۲)خیر ☐

Q2- به نظر شما خوردن شیر محلی خام (غیر حرارت دیده) منجر به ایجاد بیماری می شود؟ ۱) بلی☐ ۲) خیر☐

Q3- به نظر شما آیا نیازی است تا شیر محلی خام را قبل از مصرف جوشاند؟ ۱)بلی ☐ ۲) خیر☐

Q4- به نظر شما میوه نشسته ولی پوست کنده باعث ایجاد مسمومیت می شود؟ ۱)بلی☐ ۲) خیر☐

Q5- به نظر شما منجمد کردن باعث از بین رفتن باکتری ها می شود؟ ۱) بلی ☐ ۲)خیر☐

Q6- در کدامیک از موارد زیر باید دست ها را بشوییم. لطفا علامت بزنید

1. قبل از خوردن غذا ☐
2. قبل از آماده کردن مواد غذایی☐
3. بعد از دست زدن به گوشت و تخم مرغ خام☐
4. بعد از شکستن تخم مرغ☐
5. بعد از رفتن از دستشویی☐

Q7- بنظر شما خوردن غذاهایی که در ترکیبات آن از تخم مرغ خام استفاده می شود باعث ایجاد مسمومیت غذایی می شود؟

۱) بلی☐ ۲) خیر ☐

Q8- نیم پز کردن تخم مرغ باعث از بین رفتن باکترهای آن می شود؟ ۱) بلی☐ ۲) خیر☐

درست یا نادرست بودن جملات زیر را مشخص نمایید.

Q9- حرارت دادن و گرم کردن غذا باعث از بین رفتن تمام میکروب های آن می شود. ۱)درست☐ ۲) نادرست☐

Q10- غذاهایی که کامل پخته می شوند هیچگونه میکروب های مسمومیت زا ندارند. ۱)درست ☐ ۲) نادرست☐

Q11- برای نگهداری مواد غذایی پخته شده نیازی به نگهداری آنها در یخچال نیست. ۱)درست ☐ ۲) نادرست☐

Q12- غذاهای پخته شده را حداکثر تا چه مدت می توان در دمای اتاق نگهداری کرد؟

۱) ۲ ساعت☐ ۲) ٤ ساعت☐ ۳) ۱۲ ساعت☐ ٤) ۱ روز☐

Q13- کدامیک از مواد غذایی زیر راحت تر فاسد می شوند؟ (می توانید به بیش از یک مورد علامت بزنید)

۱) شیر☐ ۲) برنج☐ ۳) گوشت ☐ ٤) نان ☐ ۵)همه موارد☐
