# Supplementary material for: Lifestyle patterns and their nutritional, socio-demographic and psychological determinants in a community-based study: A mixed approach of latent class and factor analyses
Source: PLoS One. 2020 Jul 23;15(7):e0236242. doi: 10.1371/journal.pone.0236242 (PMC7377498; doi:10.1371/journal.pone.0236242)
Supplement: S5 File — (PDF) [file pone.0236242.s006.pdf]

GTSS

GLOBAL TOBACCO SURVEILLANCE SYSTEM

Tobacco Questions for Surveys

A Subset of Key Questions from the Global Adult Tobacco Survey (GATS)

2nd Edition

DEPARTMENT OF HEALTH & HUMAN SERVICES · USA

CDC
CENTERS FOR DISEASE CONTROL AND PREVENTION

World Health Organization

GTSS

GLOBAL TOBACCO SURVEILLANCE SYSTEM

Tobacco Questions for Surveys

A Subset of Key Questions from the Global Adult Tobacco Survey (GATS)

2nd Edition

Suggested Citation:

Global Adult Tobacco Survey Collaborative Group. *Tobacco Questions for Surveys: A Subset of Key Questions from the Global Adult Tobacco Survey (GATS), 2nd Edition*. Atlanta, GA: Centers for Disease Control and Prevention, 2011.

Contents

Section	Page
Global Tobacco Surveillance System	v
Preface	1
1. Why Use Standardized Questions in Tobacco Surveys?	2
2. Overview of the Tobacco Questions	3
3. Measuring Tobacco Smoking Prevalence	6
4. Monitor: Tobacco Smoking Consumption	8
5. Monitor: Smokeless Tobacco Use	10
6. Protect: Exposure to Secondhand Smoke	12
7. Offer: Cessation	14
8. Warn: Anti-Cigarette Information	16
9. Enforce: Cigarette Advertisements	20
10. Raise: Economics	22
11. Analysis of Tobacco Questions	24
Appendix A: GATS Overview	35
Appendix B: GATS Questionnaire Development Background	37
Appendix C: Question Mapping to GATS	39
Acknowledgements	40
Contact Information	41

Tables

Table		Page
Table 2-1.	Measuring Tobacco Smoking Prevalence	3
Table 2-2.	Measuring Key Aspects of Tobacco Surveillance	4
Table 11-1.	Detailed Smoking Status by Gender	24
Table 11-2.	Current Smokers of Various Smoked Tobacco Products, by Selected Demographic Characteristics	25
Table 11-3.	Cigarettes Smoked per Day Among Daily Cigarette Smokers, by Selected Demographic Characteristics	26
Table 11-4.	Detailed Smokeless Tobacco Use Status by Gender	27
Table 11-5.	Exposure to Tobacco Smoke at Home, by Smoking Status and Selected Demographic Characteristics	28
Table 11-6.	Exposure to Tobacco Smoke at Indoor Work Areas, by Smoking Status and Selected Demographic Characteristics	29
Table 11-7.	Current Smokers who Made a Quit Attempt and Received Health Care Provider Assistance in the Past 12 Months, by Selected Demographic Characteristics	30
Table 11-8.	Noticing Anti-Cigarette Smoking Information During the Last 30 Days in Newspapers or Magazines and Television, by Smoking Status and Selected Demographic Characteristics	31
Table 11-9.	Current Smokers who Noticed Health Warnings on Cigarette Packages and Considered Quitting Because of the Warnings During the Last 30 Days, by Selected Demographic Characteristics	32
Table 11-10.	Noticing Cigarette Advertising During the Last 30 Days in Various Places, by Selected Demographic Characteristics	33
Table 11-11.	Average Amount Spent on a Pack of Cigarettes and Cost of 100 Packs of Cigarettes as a Percentage of Gross Domestic Product (GDP) per Capita	34

GLOBAL TOBACCO SURVEILLANCE SYSTEM

In 1998, the World Health Organization (WHO), the Centers for Disease Control and Prevention (CDC), and partners initiated the Global Tobacco Surveillance System (GTSS) to assist countries in establishing tobacco control surveillance and monitoring programs. GTSS includes collection of data through three school-based surveys: the Global Youth Tobacco Survey (GYTS), the Global School Personnel Survey (GSPS), and the Global Health Professions Student Survey (GHPSS), and one household survey: the Global Adult Tobacco Survey (GATS). GTSS provides a consistent framework for surveillance including standard sampling procedures, core questionnaire items, training in field procedures, data analysis, and consistent reporting across all participating countries. GTSS also enhances the role of government and the nongovernmental sector in surveillance, monitoring of tobacco use and key tobacco control indicators, and policy and program interventions. The synergy between countries passing tobacco control laws, regulations or decrees, ratifying and complying with the WHO Framework Convention on Tobacco Control, and conducting GTSS surveys offers opportunities to develop, implement, and evaluate comprehensive tobacco control policies.

GTSS reflects WHO's, CDC's and partners' ongoing efforts to establish tobacco control surveillance and monitoring programs to track trends over time in tobacco-related knowledge, attitudes, behaviors and environmental influences.

Preface

This document *Tobacco Questions for Surveys* has been prepared for surveys that want to include questions on tobacco. If national and sub-national surveys use the questions and response categories in this guide, this will help to improve survey comparability over time and harmonize them with international tobacco surveillance and monitoring activities.

The survey questions in this guide are taken from the Global Adult Tobacco Survey (GATS) questionnaire. GATS was developed to provide a global standard protocol for consistent monitoring of adult tobacco use. GATS is designed to produce national and sub-national estimates on tobacco use, exposure to secondhand smoke, and quit attempts among adults across countries and indirectly measure the impact of tobacco control and prevention initiatives. GATS is intended to assist countries to design, implement and evaluate tobacco control and prevention programs. GATS is a nationally representative household survey of all non-institutionalized men and women aged 15 years and older. It uses a standard and consistent core questionnaire, sample design, and data collection and management techniques that were reviewed and approved by the review committees comprised of experts from developed and developing countries. A detailed overview of GATS can be found in ***Appendix A***. The GATS Core Questionnaire was constructed by tobacco control and survey design experts and has undergone rigorous development and testing, which is outlined in ***Appendix B***. The questions in this guide are mapped to the GATS Core Questionnaire in ***Appendix C***.

In order to maintain consistency and comparability in monitoring tobacco use, a standard set of survey questions on tobacco use should be implemented across various surveys, and should be periodically monitored. Given this important need, GATS partners have created the *Tobacco Questions for Surveys* booklet that includes a subset of key survey questions from the GATS for other surveys to include. Surveys can select indicators and corresponding questions from this list to include based on their particular focus or priority, or incorporate the complete set if appropriate.

This guide:

- Explains why it is important to adopt a standard set of tobacco questions in surveys.
- Introduces the set of questions on tobacco and the corresponding analysis indicators including:
  - Tobacco smoking prevalence questions, which are essential in monitoring smoking rates.
  - Additional questions covering key aspects of tobacco use and policies.
- Provides definitions and instructions for administering the questions.
- Explains how survey data from the questions are used to estimate key tobacco indicators.

1. Why Use Standardized Questions in Tobacco Surveys?

Tobacco use is a major preventable cause of premature death and disease worldwide. Just over five million people die each year due to tobacco-related illnesses – a figure expected to increase to more than eight million a year by 2030. Unless the current trend is changed, the vast majority of these deaths are projected to occur in the developing world.

The WHO Framework Convention on Tobacco Control (WHO FCTC) was developed in response to the globalization of the tobacco epidemic. The WHO FCTC and its guidelines provide the foundation for countries to implement and manage tobacco control, and sets the baseline for reducing both demand for and supply of tobacco.

To assist countries in meeting the WHO FCTC requirements, WHO introduced MPOWER, a package of selected demand reduction measures contained in the WHO FCTC. (Further information about WHO's Tobacco Free Initiative can be found at http://www.who.int/tobacco.)

Monitor tobacco use and prevention policies
Protect people from tobacco smoke
Offer help to quit tobacco use
Warn about the dangers of tobacco
Enforce bans on tobacco advertising, promotion, and sponsorship
Raise taxes on tobacco.

One of the essential components of a comprehensive global tobacco control effort is an efficient and systematic surveillance mechanism to monitor the epidemic. This is also emphasized by Article 20 (Research, surveillance and exchange of information) and Article 21 (Reporting and exchange of information) of the WHO FCTC. In order to maintain consistency and comparability in monitoring tobacco use, a standard set of tobacco use survey questions should be implemented across various surveillance activities. The Global Adult Tobacco Survey (GATS) was developed to meet this need by generating comparable data within and across countries.

The *Tobacco Questions for Surveys* guide has been created for countries that are not implementing a full GATS. This guide includes a recommended subset of key GATS questions that can be used as a standalone module or included in other existing surveys.

We are hopeful that these survey questions will become the worldwide standard, greatly enhancing the capacity of all stakeholders for monitoring and comparing trends in tobacco use and tobacco control interventions. With the expanded use of these standardized questions, the pool of reliable data will increase, hopefully leading to quality estimates at country, regional and global levels.

2. Overview of the Tobacco Questions

The survey questions in this guide assess key aspects of tobacco use surveillance. The questions are not exhaustive, but serve as a primary set to include in multiple risk factor surveys. Surveys can select particular questions and corresponding analysis indicators from this set or incorporate all of the questions if suitable. This structure was developed so that surveys which do not want to (or cannot) add all of the set of questions, could pick and choose from the various analysis indicators and questions based on their needs and tobacco control situations.

There are three basic questions that measure tobacco smoking prevalence. These questions are the highest priority measures and should be included in all surveys that measure tobacco use. Thus, for surveys with only a few question spots available, these questions should be incorporated. **Table 2-1** below provides a summary description of these three tobacco smoking prevalence questions and corresponding analysis indicators.

The three tobacco smoking prevalence questions should be included for all surveys that measure tobacco use. Surveys can then select additional questions covering key topics as appropriate.

Table 2-1. Measuring Tobacco Smoking Prevalence

	Tobacco Topic	Indicator Name and Description
Monitor	**Q1. Current tobacco smoking status**<br><br>**Q2a. Past daily smoking status**<br>(for current less than daily smokers)<br><br>**Q2b. Past smoking status**<br>(for current non-smokers)	**Current Tobacco Smokers**<br>Percentage of respondents who currently smoke tobacco.<br><br>**Current Daily Tobacco Smokers**<br>Percentage of respondents who currently smoke tobacco daily.<br><br>**Former Daily Tobacco Smokers (Among All Adults)**<br>Percentage of respondents who are ever daily tobacco smokers and currently do not smoke tobacco.<br><br>**Former Daily Tobacco Smokers (Among Ever Daily Smokers)**<br>Percentage of ever daily tobacco smokers who currently do not smoke tobacco.

The other questions in this guide cover additional key topics related to tobacco use and policies including cigarette consumption, smokeless tobacco, secondhand smoke exposure, cessation, anti-cigarette information, cigarette advertising, and economics. **Table 2-2** (on the following pages) provides a summary description of these tobacco questions and corresponding analysis indicators. The questions are grouped according to the MPOWER classification theme. Note that for surveys that want to include some or all of these questions, the three tobacco smoking prevalence questions (Q1, Q2a, and Q2b discussed above) need to be included as well, for proper survey administration and analysis indicator construction.

All of the questions are described in detail in the subsequent sections. This includes: question wording, response options, purpose of including question, instructions for administration, skip logic instructions, definitions of terms, and construction of analysis indicators. The last section of this guide provides example tables for reporting the indicators.

Note that the format of the questions and corresponding details are specific to GATS. This includes standard questionnaire administration conventions, such as question and response category text written in all uppercase letters is not to be read out loud to the respondents, and underlined text indicates words to be emphasized. Also note that GATS is conducted using in-person, interviewer administered interviews (currently using electronic data collection procedures). The questions in this guide can certainly be adapted for other survey modes of administration (e.g., telephone, paper and pencil).

Table 2-2. Measuring Key Aspects of Tobacco Surveillance

	Tobacco Topic	Indicator Name and Description
**Monitor**	**Q3. Number of tobacco products smoked per day**	**Current [*Product*] Smokers**<br>Percentage of respondents who currently smoke [*product*].<br><br>**Cigarettes Smoked per Day**<br>Average number of cigarettes smoked per day (of daily cigarette smokers)
**Monitor**	**Q4. Current smokeless tobacco use**<br><br>**Q5a. Past daily smokeless use**<br>(for current less than daily users)<br><br>**Q5b. Past smokeless use**<br>(for current non-users)	**Current Smokeless Tobacco Users**<br>Percentage of respondents who currently use smokeless tobacco.<br><br>**Current Daily Smokeless Tobacco Users**<br>Percentage of respondents who currently use smokeless tobacco daily.<br><br>**Former Daily Smokeless Tobacco Users (Among All Adults)**<br>Percentage of respondents who are ever daily smokeless tobacco users and currently do not use smokeless tobacco.<br><br>**Former Daily Smokeless Tobacco Users (Among Ever Daily Users)**<br>Percentage of ever daily smokeless tobacco users who currently do not use smokeless tobacco.
**Protect**	**Q6. Frequency of anyone smoking at home**	**Exposure to Secondhand Smoke at Home**<br>Percentage of respondents who report that smoking occurs inside their home.
	**Q7. Currently work outside home**<br><br>**Q8. Work indoor/outdoor**<br><br>**Q9. Anyone smoke at work during the past 30 days**	**Exposure to Secondhand Smoke at Work**<br>Percentage of indoor workers who were exposed to tobacco smoke at work in the past 30 days.

	Tobacco Topic	Indicator Name and Description
Offer	**Q10. Tried to quit in past 12 months**	**Smoking Quit Attempt in the Past 12 Months**<br>Percentage of current tobacco smokers who tried to quit during the past 12 months.
	**Q11. Visiting a doctor in past 12 months**<br><br>**Q12. Receiving advice to quit smoking from doctor**	**Health Care Provider's Advice to Quit Smoking Tobacco**<br>Percentage of current tobacco smokers who visited a doctor or health care provider during the past 12 months and were advised to quit smoking tobacco.
Warn	**Q13. Noticing anti-cigarette information in newspapers or magazines**	**Awareness of Anti-Cigarette Information in Newspapers/ Magazines**<br>Percentage of respondents who have noticed information about the dangers of smoking cigarettes or that encourages quitting in newspapers or magazines in the last 30 days.
	**Q14. Noticing anti-cigarette information on television**	**Awareness of Anti-Cigarette Information on Television**<br>Percentage of respondents who have noticed information about the dangers of smoking cigarettes or that encourages quitting on television in the last 30 days.
	**Q15. Noticing health warnings on cigarette packs**	**Noticing Health Warning Labels on Cigarette Packages**<br>Percentage of current smokers who noticed health warnings on cigarette packages in the last 30 days.
	**Q16. Thinking about quitting because of health warnings**	**Thinking of Quitting Because of Health Warning Labels on Cigarette Packages**<br>Percentage of current tobacco smokers who reported thinking about quitting smoking in the last 30 days because of the warning labels on cigarette packages.
Enforce	**Q17. Noticing cigarette advertisements in stores**	**Awareness of Cigarette Advertising in Stores**<br>Percentage of respondents who have noticed any advertisements or signs promoting cigarettes in stores where cigarettes are sold in the last 30 days.
	**Q18. Noticing cigarette promotions**	**Awareness of Specific Types of Cigarette Promotions**<br>Percentage of respondents who noticed [*free samples of cigarettes, cigarettes at sales prices, coupons for cigarettes, free gifts or discounts on other products when buying cigarettes, clothing or other items with a cigarette brand name or logo, cigarette promotions in the mail*] in the last 30 days.
Raise	**Q19. Last cigarette purchase – quantity**<br><br>**Q20. Last cigarette purchase – cost**	**Cost of Manufactured Cigarettes**<br>Average amount spent on a pack of manufactured cigarettes (in local currency).<br><br>**Cigarette Affordability**<br>Average cost of 100 packs of manufactured cigarettes as a percentage of Gross Domestic Product (GDP) per capita.

3. Measuring Tobacco Smoking Prevalence

The highest priority indicators are constructed from the three questions which assess tobacco smoking prevalence. The first question (Q1) measures current use, the second question (Q2a) measures past daily use for current less than daily smokers, and the third question (Q2b) measures past use for current non-smokers. It is imperative that these questions be included in any survey that wants to examine basic tobacco smoking prevalence rates.

Q1. Current Tobacco Smoking Status

Do you currently smoke tobacco on a daily basis, less than daily, or not at all?

DAILY .... ☐ 1 > END SECTION
LESS THAN DAILY .... ☐ 2 > ASK Q2a
NOT AT ALL .... ☐ 3 > ASK Q2b
DON'T KNOW .... ☐ 7 > END SECTION

Q2a. Past Daily Smoking Status

Have you smoked tobacco daily in the past?

YES .... ☐ 1 > END SECTION
NO .... ☐ 2 > END SECTION
DON'T KNOW .... ☐ 7 > END SECTION

Q2b. Past Smoking Status

In the past, have you smoked tobacco on a daily basis, less than daily, or not at all?

INTERVIEWER: IF RESPONDENT HAS DONE BOTH "DAILY" AND "LESS THAN DAILY" IN THE PAST, CHECK "DAILY"

DAILY .... ☐ 1
LESS THAN DAILY .... ☐ 2
NOT AT ALL .... ☐ 3
DON'T KNOW .... ☐ 7

Purpose

Determines the current and past tobacco smoking status of the respondents.

Instructions

Q1: Ask question and record only one answer. (DON'T KNOW should not be read to the respondent.) If the answer is DAILY or DON'T KNOW, Q2a and Q2b will not be asked.

Q2a: This question will only be administered if the respondent says he/she currently smokes less than daily in question Q1 (Q1=2). Ask question and select only one answer. (DON'T KNOW should not be read to the respondent.)

Q2b: This question will only be administered if the respondent says he/she does not currently smoke at all in question Q1 (Q1=3). Ask question and select only one answer. (DON'T KNOW should not be read to the respondent.) Note that if a respondent says he/she smoked daily and less than daily in the past, select the answer for DAILY.

Definitions

Daily means smoking at least one tobacco product every day or nearly every day over a period of a month or more.

Rare instances of smoking or experimental smoking (tried once or twice in lifetime) should be counted in the NOT AT ALL category.

Indicator 1

Current Tobacco Smokers: Percentage of respondents who currently smoke tobacco.

Numerator: Number of current daily and less than daily tobacco smokers.

Denominator: Total number of respondents surveyed. ("Don't know" responses are excluded.)

Indicator 2

Current Daily Tobacco Smokers: Percentage of respondents who currently smoke tobacco daily.

Numerator: Number of current daily tobacco smokers.

Denominator: Total number of respondents surveyed. ("Don't know" responses are excluded.)

Indicator 3

Former Daily Tobacco Smokers (Among All Adults): Percentage of respondents who are ever daily tobacco smokers and currently do not smoke tobacco.

Numerator: Number of ever daily tobacco smokers who currently do not smoke tobacco.

Denominator: Total number of respondents surveyed. ("Don't know" responses are excluded.)

Indicator 4

Former Daily Tobacco Smokers (Among Ever Daily Smokers): Percentage of ever daily tobacco smokers who currently do not smoke tobacco.

Numerator: Number of ever daily tobacco smokers who currently do not smoke tobacco.

Denominator: Number of ever daily tobacco smokers. ("Don't know" responses are excluded.)

4. Monitor: Tobacco Smoking Consumption

Question 3 asks about the amount of tobacco being smoked by current smokers. This question (with item parts) measures usage of cigarettes and other specific tobacco products.

Q3. Number of Tobacco Products Smoked Per Day

On average, how many of the following products do you currently smoke each (day/week)? Also, let me know if you smoke the product, but not every (day/week).

INTERVIEWER: IF RESPONDENT REPORTS SMOKING THE PRODUCT BUT NOT EVERY (DAY/WEEK), ENTER 888

a. Manufactured cigarettes?	☐☐☐	PER DAY/WEEK	INT: VERIFY THIS IS # OF CIGARETTES, NOT PACKS
b. Hand-rolled cigarettes?	☐☐☐	PER DAY/WEEK	
c. Kreteks?	☐☐☐	PER DAY/WEEK	
d. Pipes full of tobacco?	☐☐☐	PER DAY/WEEK	
e. Cigars, cheroots, or cigarillos?	☐☐☐	PER DAY/WEEK	
f. Number of water pipe sessions?	☐☐☐	PER DAY/WEEK	
g. Any others?	☐☐☐	PER DAY/WEEK	Specify: ______

Purpose

Determines the number of tobacco products that the respondent smokes each day or week. (Note that the tobacco products should be adjusted for the specific survey/country.)

Instructions

Administered to respondents who are currently smokers (Q1=1 or 2). Ask stem question in Q3 and each item part (a through g). For current daily smokers (Q1=1), the question should ask about daily use (number per day). For current less than daily smokers (Q1=2), the question should ask about weekly use (number per week).

If the respondent answers that he/she smokes one of the products but less than daily/weekly, enter "888".

If the respondent reports an answer in packs, the interviewer needs to record the actual number of the tobacco product of interest, rather than the number of packs. This is especially important for item part a (Manufactured cigarettes) as respondents might answer in number of packs of cigarettes.

Indicator 1

Current [*Product*] Smokers: Percentage of respondents who currently smoke [*product*].

Numerator: Number of current daily and less than daily [*product*] smokers.

Denominator: Total number of respondents surveyed.

Indicator 2

Number of Cigarettes Smoked Per Day (of daily cigarette smokers).

Numerator: Daily cigarette smokers reporting an average of [less than 5, 5-9, 10-14, 15-24, 25+] cigarettes per day.

Denominator: Daily cigarette smokers.

5. Monitor: Smokeless Tobacco Use

Questions 4, 5a, and 5b measure the prevalence of smokeless tobacco use. These questions mirror the smoking tobacco prevalence questions (Q1, Q2a, and Q2b).

Q4. Current Smokeless Tobacco Use

Do you currently use smokeless tobacco on a daily basis, less than daily, or not at all?

DAILY	☐ 1 > END SECTION
LESS THAN DAILY	☐ 2 > ASK Q5a
NOT AT ALL	☐ 3 > ASK Q5b
DON'T KNOW	☐ 7 > END SECTION

Q5a. Past Daily Smokeless Tobacco Use

Have you used smokeless tobacco daily in the past?

YES	☐ 1 > END SECTION
NO	☐ 2 > END SECTION
DON'T KNOW	☐ 7 > END SECTION

Q5b. Past Smokeless Tobacco Use

In the past, have you used smokeless tobacco on a daily basis, less than daily, or not at all?

INTERVIEWER: IF RESPONDENT HAS DONE BOTH "DAILY" AND "LESS THAN DAILY" IN THE PAST, CHECK "DAILY"

DAILY	☐ 1
LESS THAN DAILY	☐ 2
NOT AT ALL	☐ 3
DON'T KNOW	☐ 7

Purpose

Determines the current and past smokeless tobacco use status of the respondents.

Instructions

Q4: Ask question and record only one answer. (DON'T KNOW should not be read to the respondent.) If the answer is DAILY or DON'T KNOW, Q5a and Q5b will not be asked.

Q5a: This question will only be administered if the respondent says he/she currently uses smokeless tobacco less than daily in question Q4 (Q4=2). Ask question and select only one answer. (DON'T KNOW should not be read to the respondent.)

Q5b: This question will only be administered if the respondent says he/she does not use smokeless tobacco at all in question Q4 (Q4=3). Ask question and select only one answer. (DON'T KNOW should not be read to the respondent.) Note that if a respondent says he/she used smokeless tobacco daily <u>and</u> less than daily in the past, select the answer for DAILY.

Definitions

Daily means using smokeless tobacco at least one time every day or nearly every day over a period of a month or more.

Rare instances of using smokeless tobacco or experimental use (tried once or twice in lifetime) should be counted in the NOT AT ALL category.

Indicator 1

Current Smokeless Tobacco Users: Percentage of respondents who currently use smokeless tobacco.

Numerator: Number of current daily and less than daily smokeless tobacco users.

Denominator: Total number of respondents surveyed. ("Don't know" responses are excluded.)

Indicator 2

Current Daily Smokeless Tobacco Users: Percentage of respondents who currently use smokeless tobacco daily.

Numerator: Number of current daily smokeless tobacco users.

Denominator: Total number of respondents surveyed. ("Don't know" responses are excluded.)

Indicator 3

Former Daily Smokeless Tobacco Users (Among All Adults): Percentage of respondents who are ever daily smokeless tobacco users and currently do not use smokeless tobacco.

Numerator: Number of ever daily smokeless tobacco users who currently do not use smokeless tobacco.

Denominator: Total number of respondents surveyed. ("Don't know" responses are excluded.)

Indicator 4

Former Daily Smokeless Tobacco Users (Among Ever Daily Users): Percentage of ever daily smokeless tobacco users who currently do not use smokeless tobacco.

Numerator: Number of ever daily smokeless tobacco users who currently do not use smokeless tobacco.

Denominator: Number of ever daily smokeless tobacco users. ("Don't know" responses are excluded.)

6. Protect: Exposure to Secondhand Smoke

These questions measure secondhand smoke exposure in the home (Q6) and in the workplace (Q7, Q8, Q9).

Q6. Frequency of Smoking in the Home

How often does anyone smoke inside your home? Would you say daily, weekly, monthly, less than monthly, or never?

DAILY .... ☐ 1

WEEKLY .... ☐ 2

MONTHLY .... ☐ 3

LESS THAN MONTHLY .... ☐ 4

NEVER .... ☐ 5

DON'T KNOW .... ☐ 7

Purpose

Determines the frequency of anyone smoking inside the respondent's home.

Instructions

Ask question and select only one answer. The respondent should select one of the response categories that comes closest to his/her situation. (DON'T KNOW should not be read to the respondent.)

Definitions

This question is asking about inside the respondent's home. This only includes enclosed areas of the home – the respondent should not include areas outside of the home including patios, porches, etc. that are not fully enclosed.

Indicator

Exposure to Secondhand Smoke at Home: Percentage of respondents who report that smoking occurs inside their home.

Numerator: Number of respondents who reported that smoking occurs inside their home on daily, weekly, or monthly basis.

Denominator: Total number of respondents surveyed. ("Don't know" responses are excluded.)

Q7. Current Working Location

Do you currently work outside of your home?

YES ....☐ 1 > ASK Q8

NO/DON'T WORK ....☐ 2 > END SECTION

Q8. Currently Working Indoors or Outdoors

Do you usually work indoors or outdoors?

INDOORS ....☐ 1 > ASK Q9

OUTDOORS..☐ 2 > END SECTION

BOTH...☐ 3 > ASK Q9

Q9. Smoking at the Workplace

During the past 30 days, did anyone smoke in indoor areas where you work?

YES ....☐ 1

NO ....☐ 2

DON'T KNOW ....☐ 7

Purpose

These questions determine if anyone smoked in indoor areas at the respondent's workplace. The first question (Q7) determines whether the respondent works outside of the home. The second question (Q8) determines if the respondent usually works inside or outside. The third question (Q9) determines if anyone smoked in indoor areas where the respondent worked in the past 30 days.

Instructions

Q7: Ask question and select only one answer. If the respondent says he/she does not currently work, NO/DON'T WORK should be selected. If the answer is NO or DON'T WORK, questions Q8 and Q9 will not be asked.

Q8: Ask question and select only one answer. If the respondent says he/she works both indoors and outdoors, select BOTH. If the answer is OUTDOORS, question Q9 will not be asked.

Q9: Ask question and select only one answer. The respondent should answer yes if he/she saw somebody smoke or smelled the smoke. (DON'T KNOW should not be read to the respondent.)

Indicator

Exposure to Secondhand Smoke at Work: Percentage of indoor workers who were exposed to tobacco smoke at work in the past 30 days.

Numerator: Number of respondents who reported being exposed to smoke in indoor areas at work during the past 30 days.

Denominator: Number of respondents who work outside of the home who usually work indoors or both indoors and outdoors. ("Don't know" responses to Q9 are excluded.)

7. Offer: Cessation

Two concepts are measured with these questions: 1) Attempt of current smokers to quit (Q10), and 2) Receiving advice from healthcare providers to quit (Q11, Q12).

Q10. Attempting to Quit Smoking

During the past 12 months, have you tried to stop smoking?

YES .... ☐ 1

NO .... ☐ 2

Purpose

Determines if current smokers have tried to quit in the last 12 months.

Instructions

Administered to current smokers (Q1=1 or 2). Ask question and select only one answer.

Indicator

Smoking Quit Attempt in the Past 12 Months: Percentage of current tobacco smokers who have tried to quit during the past 12 months.

Numerator: Current tobacco smokers who tried to quit during the past 12 months.

Denominator: Current tobacco smokers.

Q11. Visiting a Doctor

Have you visited a doctor or other health care provider in the past 12 months?

YES .... ☐ 1 > ASK Q12

NO .... ☐ 2 > SKIP Q12

Q12. Receiving Cessation Advice from Doctor

During any visit to a doctor or health care provider in the past 12 months, were you advised to quit smoking tobacco?

YES .... ☐ 1

NO .... ☐ 2

Purpose

Q11 determines if the respondent went to a doctor or health care provider in the past 12 months for his/her health and if yes, Q12 determines whether the respondent was advised by a doctor or health care provider to quit smoking tobacco.

Instructions

Both Q11 and Q12 are only administered to current smokers (Q1=1 or 2).

Q11: Ask question and select only one answer.

Q12: Administered only if Q11=1. Ask question and select only one answer.

Indicator

Health Care Provider's Advice to Quit Smoking Tobacco: Percentage of current tobacco smokers who visited a doctor or health care provider during the past 12 months and were advised to quit smoking tobacco.

Numerator: Number of current tobacco smokers who were advised to quit smoking during a visit to a healthcare provider within the past 12 months.

Denominator: Number of current tobacco smokers who visited a healthcare provider in the past 12 months.

8. Warn: Anti-Cigarette Information

There are four important measures that are included in this section: 1) Awareness of anti-cigarette information in newspapers or magazines (Q13), 2) Awareness of anti-cigarette information in television (Q14), 3) Noticing health warnings on cigarette packs (Q15), and 4) Current smokers thinking about quitting because of the health warnings on cigarette packs (Q16).

Q13. Noticing Anti-Cigarette Information in Newspapers/Magazines

In the last 30 days, have you noticed information about the dangers of smoking cigarettes or that encourages quitting in newspapers or in magazines?

YES .... ☐ 1

NO .... ☐ 2

NOT APPLICABLE .... ☐ 7

Purpose

Determines if the respondent noticed, in the past 30 days, any information in newspapers or magazines about the dangers of smoking cigarettes or that encourages quitting.

Instructions

Ask question and select only one answer. If the respondent says he/she did not read any newspapers or magazines in the past 30 days, select the NOT APPLICABLE category but do not offer this category to the respondents.

Indicator

Awareness of Anti-Cigarette Smoking Information in Specific Channels: Percentage of respondents who have noticed information about the dangers of smoking cigarettes or that encourages quitting in newspapers or magazines in the last 30 days. (It is recommended that this indicator be reported for the overall population and separately among current tobacco smokers and non-smokers.)

Numerator: Number of respondents who have noticed information about the dangers of smoking cigarettes or that encourages quitting in newspapers or magazines in the last 30 days.

Denominator: Total number of respondents surveyed. ("Not applicable" responses are included.)

Q14. Noticing Anti-Cigarette Information on Television

In the last 30 days, have you noticed information about the dangers of smoking cigarettes or that encourages quitting on television?

YES .... ☐ 1

NO .... ☐ 2

NOT APPLICABLE .... ☐ 7

Purpose

Determines if the respondent noticed, in the past 30 days, any information on television about the dangers of smoking cigarettes or that encourages quitting.

Instructions

Ask question and select only one answer. If the respondent says he/she did not watch any television in the past 30 days, select the NOT APPLICABLE category but do not offer this category to the respondents.

Indicator

Awareness of Anti-Cigarette Smoking Information in Specific Channels: Percentage of respondents who have noticed information about the dangers of smoking cigarettes or that encourages quitting on television in the last 30 days. (It is recommended that this indicator be reported for the overall population and separately among current tobacco smokers and non-smokers).

Numerator: Number of respondents who have noticed information about the dangers of smoking cigarettes or that encourages quitting on television in the last 30 days.

Denominator: Total number of respondents surveyed. ("Not applicable" responses are included.)

Q15. Noticing Health Warnings on Cigarette Packs

In the last 30 days, did you notice any health warnings on cigarette packages?

YES .... ☐ 1

NO .... ☐ 2 > END SECTION

DID NOT SEE ANY CIGARETTE PACKAGES........................ ☐ 3 > END SECTION

Purpose

Determines if current smokers noticed health warnings on cigarette packages in the past 30 days.

Instructions

Administered if the respondent is a current smoker (Q1=1 or 2). Ask question and select only one answer. Do not read response categories.

Indicator

Noticing Health Warning Labels on Cigarette Packages: Percentage of current smokers who noticed health warnings on cigarette packages in the last 30 days.

Numerator: Number of current smokers who noticed health warnings on cigarette packages in the last 30 days.

Denominator: Number of current smokers.

Q16. Thinking About Quitting Because of Health Warnings on Cigarette Packs

In the last 30 days, have warning labels on cigarette packages led you to think about quitting?

YES .... ☐ 1

NO .... ☐ 2

DON'T KNOW .... ☐ 7

Purpose

Determines if current smokers thought about quitting because of noticing warning labels.

Instructions

Administered if the respondent is a current smoker (Q1=1 or 2) and noticed the health warnings on cigarette packages in the past 30 days (Q15=1). Ask question and select only one answer.

Indicator

Thinking of Quitting Because of Health Warning Labels on Cigarette Packages: Percentage of current tobacco smokers who reported thinking about quitting smoking in the last 30 days because of the warning labels on cigarette packages.

Numerator: Number of current smokers who thought about quitting smoking in the last 30 days because of the warning labels on cigarette packages.

Denominator: Number of current smokers. ("Don't know" responses are included.)

9. Enforce: Cigarette Advertisements

These questions measure respondents' exposure to cigarette advertising in stores (Q17) and various cigarette promotions (Q18).

Q17. Cigarette Advertising in Stores

In the last 30 days, have you noticed any advertisements or signs promoting cigarettes in stores where cigarettes are sold?

YES .... ☐ 1

NO .... ☐ 2

NOT APPLICABLE .... ☐ 7

Purpose

Determines if the respondent has, in the past 30 days, seen advertisements or signs promoting cigarettes in stores where cigarettes are sold.

Instructions

Ask question and select only one answer. If the respondent says he/she did not go to any stores where cigarettes are sold in the past 30 days, select the NOT APPLICABLE category but do not offer this category to the respondents.

Indicator

Awareness of Cigarette Advertising in Specific Channels: Percentage of respondents who have noticed any advertisements or signs promoting cigarettes in stores in the last 30 days. (It is recommended that this indicator be reported for the overall population and separately among current tobacco smokers and non-smokers.)

Numerator: Number of respondents who have noticed any advertisements or signs promoting cigarettes in stores in the last 30 days.

Denominator: Total number of respondents surveyed. ("Not applicable" responses are included.)

Q18. Cigarette Promotions

In the last 30 days, have you noticed any of the following types of cigarette promotions?

READ EACH ITEM:	YES	NO	DON'T KNOW
	▼	▼	▼
a. Free samples of cigarettes?	☐ 1	☐ 2	☐ 7
b. Cigarettes at sale prices?	☐ 1	☐ 2	☐ 7
c. Coupons for cigarettes?	☐ 1	☐ 2	☐ 7
d. Free gifts or special discount offers on other products when buying cigarettes?	☐ 1	☐ 2	☐ 7
e. Clothing or other items with a cigarette brand name or logo?	☐ 1	☐ 2	☐ 7
f. Cigarette promotions in the mail?	☐ 1	☐ 2	☐ 7

Purpose

Q18 a through f determine if the respondent has seen various cigarette promotions in the past 30 days.

Instructions

Ask stem question and then each item part (a through f). Only repeat stem question if needed. Do not read response categories.

Note that items can be adjusted depending on the specific situation for the survey location (e.g., country).

Indicator

Awareness of Specific Types of Cigarette Promotions: Percentage of respondents who noticed [*free samples of cigarettes, cigarettes at sales prices, coupons for cigarettes, free gifts or discounts on other products when buying cigarettes, clothing or other items with a cigarette brand name or logo, cigarette promotions in the mail*] in the last 30 days. (It is recommended that this indicator be reported for the overall population and separately among current tobacco smokers and non-smokers.)

Numerator: Number of respondents who noticed [*free samples of cigarettes, cigarettes at sales prices, coupons for cigarettes, free gifts or discounts on other products when buying cigarettes, clothing or other items with a cigarette brand name or logo, cigarette promotions in the mail*] in the last 30 days.

Denominator: Total number of respondents surveyed. ("Don't know" responses are included.)

10. Raise: Economics

Data from the two questions in this section (Q19, Q20) are used with cigarette consumption data (Q3a) to create the economic indicators described below. Note that Q3a (previously detailed) must be selected along with Q19 and Q20 in order to properly administer these questions and calculate the indicators.

Q19. Last Cigarette Purchase - Quantity

The last time you bought cigarettes for yourself, how many cigarettes did you buy?

INTERVIEWER: RECORD NUMBER AND CHECK UNIT

☐☐☐

CIGARETTES .... ☐ 1

PACKS.. ☐ 2 > How many cigarettes were in each pack? ☐☐

CARTON .... ☐ 3 > How many cigarettes were in each carton? ☐☐☐

OTHER SPECIFY:_______________ ☐ 4 > How many cigarettes were in each [FILL]? ☐☐☐

NEVER BOUGHT CIGARETTES.... ☐ 5 > END SECTION

Q20. Last Cigarette Purchase - Cost

In total, how much money did you pay for this purchase?

INTERVIEWER: IF DON'T KNOW, ENTER 999

☐☐☐ [*FILL COUNTRY CURRENCY*]

Purpose

These two questions determine the quantity of cigarettes and how much money was paid the last time the respondent bought cigarettes for him/herself. Note that question Q3a is needed to administer these questions and to create the indicators.

Instructions

Both Q19 and Q20 are only administered to current smokers of manufactured cigarettes (Q1=1 or 2 AND Q3a=1-888).

Q19: Ask question and record the number and select the unit the respondent provided (cigarettes, packs, cartons, other). Specify the unit if OTHER. If the answer was reported in packs, cartons, or other, ask the respondent how many cigarettes were in each.

Q20: Ask question and enter in amount of purchase. Note that the type of currency will be adjusted for the specific survey location (e.g., country).

Indicator 1

Average Cost of a Pack of Manufactured Cigarettes (in local currency).

Calculation:

1) Using information on the number and unit of last purchase (e.g., 2 packs) and the number of cigarettes per unit (e.g., 20 cigarettes per pack), calculate the number of manufactured cigarettes bought at last purchase (2 packs x 20 cigarettes per pack = 40 cigarettes).

2) Divide the amount paid for the last purchase of manufactured cigarettes by the number of manufactured cigarettes bought at the last purchase to calculate the amount paid per cigarette (e.g., $10/40 cigarettes = $.25 per cigarette).

3) Multiply the amount paid per cigarette by 20 cigarettes/pack to calculate the amount paid per pack of manufactured cigarettes (e.g., $.25 x 20 cigarettes/pack = $5).

4) Calculate the number of manufactured cigarettes smoked per day for each individual (using Q3a).

5) Generate a new "manufactured cigarette weight", equal to the product of the individual sampling weight and the number of manufactured cigarettes smoked per day.

6) Calculate the average amount paid per pack of manufactured cigarettes across all respondents, weighted by the new "manufactured cigarette weight".

Indicator 2

Cigarette Affordability: Average cost of 100 packs of manufactured cigarettes as a percentage of Gross Domestic Product (GDP) per capita.

Calculation:

1) Use the same approach as described above to calculate the consumption-weighted average cost per pack of 20 manufactured cigarettes.

2) Multiply the average cost per pack by 100 to estimate the average cost of 100 packs.

3) Divide the average cost of 100 packs by the per capita GDP and multiply by 100.

11. Analysis of Tobacco Questions

This section provides examples of analysis table shells that serve as guidance for data analysis of the questions in this guide. Note these example tables are specific for GATS, where key GATS demographic variables (e.g., gender, urban/rural location, education) are included. Analysis tables should certainly be adjusted for the specific survey's needs.

Measuring Tobacco Smoking Prevalence

Table 11-1. Detailed Smoking Status by Gender

Smoking Status	Overall	Male	Female
		Percentage (95% CI)	
**Current tobacco smoker**			
Daily smoker			
Occasional smoker			
Occasional smoker, formerly daily			
Occasional smoker, never daily			
**Current non-smoker**			
Former smoker			
Former daily smoker			
Former occasional smoker			
Never smoker			

Table 11-1 provides a detailed description of smoking status among the population. Each of these indicators is calculated among all respondents in that group/category. All rows should have estimates, including the bolded "current tobacco smoker" and "non-smoker" rows. These two bolded estimates should add to 100%. Each level of indentation refers to subheadings that should add to the estimates above them (with the potential for rounding error). For example, the prevalence of "daily" and "occasional" smokers should add to the overall "current tobacco smoker" prevalence. Similarly, the prevalence of "occasional smoker, formerly daily" and "occasional smoker, never daily" should add to the "occasional smoker" prevalence. Many of the subcategories will almost never be used separately (e.g., "occasional smoker, formerly daily" and "occasional smoker, never daily") and might be routinely combined. However, it is recommended that for this table, the detailed smoking status categories be reported.

Table 11-2. Current Smokers of Various Smoked Tobacco Products, by Selected Demographic Characteristics

Demographic Characteristics	Any smoked tobacco product	Any cigarette[1]	Type of Cigarette		Other smoked tobacco[2]
			Manufactured	Hand-rolled	
			Percentage (95% CI)		
**Overall**					
Gender					
Male					
Female					
Age (years)					
15-24					
25-44					
45-64					
65+					
Residence					
Urban					
Rural					
Education Level[3]					
[Category 1					
Category 2					
Category 3]					

Note: Current use includes both daily and occasional (less than daily) use.
[1] Includes manufactured cigarettes, hand rolled cigarettes, and kreteks.
[2] Includes [describe products included in "other" category here].
[3] Education level is reported only among respondents 25+ years old.

Table 11-2 describes the prevalence of current tobacco smoking and current use of specific products. Countries should adapt this table to report products with significant usage here, for example bidis or water pipes. If kretek usage is reported it should be documented in the table under "Type of Cigarette" along with "Manufactured" and "Hand-rolled". The prevalence estimates include both daily and occasional (less than daily use). "Any smoked tobacco" includes any type of smoked tobacco, while "any cigarette" includes manufactured, hand-rolled, and/or kreteks, where applicable. It is important to note that the estimates reflect prevalence, not the percent distribution of product use; therefore, the denominator for each of these indicators is all respondents in each subgroup. For example, the estimate for manufactured cigarettes is the percentage of all respondents who are current smokers of manufactured cigarettes, not the percent of current smokers who smoke manufactured cigarettes. For this reason, the estimates should not be expected to add to 100%.

Table 11-3. Cigarettes Smoked per Day Among Daily Cigarette Smokers, by Selected Demographic Characteristics

Demographic Characteristics	Number of cigarettes smoked on average per day[1]					Total
	<5	5-9	10-14	15-24	≥25	
	Percentage (95% CI)					
**Overall**						100.0
Gender						
Male						100.0
Female						100.0
Age (years)						
15-24						100.0
25-44						100.0
45-64						100.0
65+						100.0
Residence						
Urban						100.0
Rural						100.0
Education Level[2]						
[Category 1						100.0
Category 2						100.0
Category 3]						100.0

[1] Among daily cigarette smokers. Cigarettes include manufactured, hand-rolled, and kreteks.
[2] Education level is reported only among respondents 25+ years old.

Table 11-3 describes the distribution of cigarette smoking consumption among daily cigarette smokers. The categories presented here should add to 100%. The average number of cigarettes smoked per day is also recommended to be reported (overall and for each of the subgroups).

Table 11-4. Detailed Smokeless Tobacco Use Status by Gender

Smoking Status	Overall	Male	Female
		Percentage (95% CI)	
**Current smokeless tobacco user**			
Daily user			
Occasional user			
Occasional user, formerly daily			
Occasional user, never daily			
**Current non-user of smokeless tobacco**			
Former user			
Former daily user			
Former occasional user			
Never smokeless user			

Table 11-4 presents data for smokeless tobacco use, which mirrors Table 11-1 for tobacco smoking.

Table 11-5. Exposure to Tobacco Smoke at Home, by Smoking Status and Selected Demographic Characteristics

Demographic Characteristics	Respondents exposed to tobacco smoke at home[1]	
	Overall	Non-smokers
	Percentage (95% CI)	
**Overall**		
Gender		
Male		
Female		
Age (years)		
15-24		
25-44		
45-59		
65+		
Residence		
Urban		
Rural		
Education Level[2]		
[Category 1		
Category 2		
Category 3]		

[1] Respondents who reported that smoking inside the home occurs daily, weekly, or monthly.
[2] Education level is reported only among respondents 25+ years old.

Table 11-5 presents the percentage of respondents who are exposed to tobacco smoke in their homes on an at least monthly basis.

Table 11-6. Exposure to Tobacco Smoke at Indoor Work Areas, by Smoking Status and Selected Demographic Characteristics

Demographic Characteristics	Respondents exposed to tobacco smoke at work[1]	
	Overall	Non-smokers
	Percentage (95% CI)	
**Overall**		
Gender		
Male		
Female		
Age (years)		
15-24		
25-44		
45-59		
65+		
Residence		
Urban		
Rural		
Education Level[2]		
[Category 1		
Category 2		
Category 3]		

[1] In the past 30 days. Among those respondents who work outside of the home who usually work indoors or both indoors and outdoors.
[2] Education level is reported only among respondents 25+ years old.

Table 11-6 provides the percentage of respondents exposed to tobacco at their workplace in the past 30 days.

Offer: Cessation

Table 11-7. Current Smokers who Made a Quit Attempt and Received Health Care Provider Assistance in the Past 12 Months, by Selected Demographic Characteristics

Demographic Characteristics	Smoking cessation and health care seeking behavior		
	Made quit attempt[1]	Visited a HCP[1,2]	Advised to quit by HCP[2,3]
		Percentage (95% CI)	
**Overall**			
Gender			
Male			
Female			
Age (years)			
15-24			
25-44			
45-64			
65+			
Residence			
Urban			
Rural			
Education Level[4]			
[Category 1			
Category 2			
Category 3]			

[1] Among current smokers.
[2] HCP = health care provider.
[3] Among current smokers who visited a HCP during the past 12 months.
[4] Education level is reported only among respondents 25+ years old.

Table 11-7 describes smoking cessation behavior, health care seeking behavior, and health care provider assistance. Note the different denominators for some of the indicators.

Table 11-8. Noticing Anti-Cigarette Smoking Information During the Last 30 Days in Newspapers or Magazines and Television, by Smoking Status and Selected Demographic Characteristics

Places	Overall	Gender		Age (years)		Residence	
		Male	Female	15-24	≥ 25	Urban	Rural
	Percentage (95% CI)						
Overall							
In newspapers or in magazines							
On television							
Current smokers[1]							
In newspapers or in magazines							
On television							
Non-smokers[2]							
In newspapers or in magazines							
On television							

[1] Includes daily and occasional (less than daily) smokers.
[2] Includes former and never smokers.

Table 11-8 describes the extent to which respondents have noticed anti-cigarette smoking information in newspapers or magazines and on television, by smoking status and suggested demographic characteristics.

Table 11-9. Current Smokers who Noticed Health Warnings on Cigarette Packages and Considered Quitting Because of the Warnings During the Last 30 Days, by Selected Demographic Characteristics

Demographic Characteristics	Current smokers[1] who…	
	Noticed health warnings on cigarette package[2]	Thought about quitting because of warning label[2]
	Percentage (95% CI)	
**Overall**		
Gender		
Male		
Female		
Age (years)		
15-24		
25-44		
45-64		
65+		
Residence		
Urban		
Rural		
Education Level[3]		
[Category 1		
Category 2		
Category 3]		

[1] Includes daily and occasional (less than daily) smokers.
[2] During the last 30 days.
[3] Education level is reported only among respondents 25+ years old.

Table 11-9 describes the percentage of current smokers who noticed health warnings on cigarette packages or thought about quitting because of the health warning. Labeling of tobacco products with health warnings is a key provision of the WHO Framework Convention on Tobacco Control (WHO FCTC). They provide a cue to help smokers quit and to counteract the marketing of tobacco products. Both of these indicators should be calculated among current smokers.

ENFORCE: CIGARETTE ADVERTISEMENTS

Table 11-10. Noticing Cigarette Advertising During the Last 30 Days in Various Places, by Selected Demographic Characteristics

Places	Overall	Gender		Age (years)		Residence	
		Male	Female	15-24	≥ 25	Urban	Rural
				Percentage (95% CI)			
Noticed advertisements in stores							
Noticed cigarette promotions							
Free samples							
Sale prices							
Coupons							
Free gifts/discounts on other products							
Clothing/item with brand name or logo							
Mail promoting cigarettes							

Table 11-10 describes the extent to which respondents have noticed cigarette advertising in stores and various cigarette promotions. It is recommended to repeat this table for current smokers and non-smokers.

Table 11-11. Average Amount Spent on a Pack of Cigarettes and Cost of 100 Packs of Cigarettes as a Percentage of Gross Domestic Product (GDP) per Capita

	**Local Currency**
Average amount spent on 20 manufactured cigarettes	XX.X
	**Overall (%)**
Cost of 100 packs of manufactured cigarettes as a percentage of per capita Gross Domestic Product (GDP)	XX.X

Table 11-11 provides two economic indicators which are calculated among current smokers who smoke manufactured cigarettes at least once per week. Both of these estimates use a weighted average cost that is equivalent to the total expenditure on manufactured cigarettes per day across the target population divided by the total daily consumption of manufactured cigarettes.

Appendix A: GATS Overview

The Global Adult Tobacco Survey (GATS) is designed to produce national and sub-national estimates on tobacco use, exposure to secondhand smoke, quit attempt among adults across countries and indirectly measure the impact of various tobacco control and prevention initiatives at country level. GATS, a component of Global Tobacco Surveillance System (GTSS), is intended to enhance the capacity of countries to design, implement, and evaluate tobacco control and prevention programs. The GTSS tracks tobacco use and tobacco control measures. In addition to GATS, GTSS consists of three school-based surveys for youth and selected adult populations: the Global Youth Tobacco Survey (GYTS), the Global School Personnel Survey (GSPS), and the Global Health Professions Student Survey (GHPSS).

GATS is a nationally representative household survey of all non-institutionalized, men and women age 15 years and older using a standard and consistent core questionnaire, sample design and data collection and management that were reviewed and approved by the review committees consisting of experts both from developed and developing countries across the world in tobacco control, survey design and methodology. Currently, data for this survey are collected by means of a face-to-face personal interview using electronic data collection procedures with the help of handheld computers. GATS uses standard best practices such as pretesting questionnaires; reviewing survey proposals; technical assistance and training on data collection and management; conducting workshops and orientations; and providing consultation and technical feedback on data analysis and reporting.

Core Questionnaire

The GATS Core Questionnaire consists of a core set of questions and a list of optional questions. Participating countries may add and/or adapt questions to the Core Questionnaire related to the country-specific situation. Once finalized, the questionnaire is translated into local languages, as applicable before the survey administration. The GATS Core Questionnaire is designed to gather data on the following topics:

- *Background characteristics*: Gender, age, education, work status, possession of household items.
- *Tobacco smoking*: Patterns of use; Former/past tobacco consumption; Age of initiation of daily smoking; Frequency of consumption of different smoked tobacco products; Nicotine dependence; Frequency of quit attempts.
- *Smokeless tobacco*: Patterns of use; Former/past tobacco consumption; Age of initiation of daily smokeless tobacco use; Frequency of consumption of different smokeless tobacco products; Nicotine dependence; Frequency of quit attempts.
- *Cessation*: Advice to quit smoking/smokeless tobacco use by health care provider; Methods used to try to stop smoking/smokeless tobacco use.
- *Secondhand smoke*: Smoking allowed in the home; Exposure to secondhand smoke at home and public places; Indoor smoking policy at work place; Knowledge of serious illness in non-smokers due to secondhand smoke.

- *Economics*: Type of tobacco product and quantity bought; Cost, brand and type of tobacco product(s); Source of tobacco products.
- *Media*: Exposure to tobacco related advertisement in various places; Exposure to sporting events connected with tobacco; Exposure to music, theatre, art or fashion events connected with tobacco; Exposure to tobacco promotion activities; Health warning labels on cigarette packages; Exposure to anti-tobacco advertising and information.
- *Knowledge, attitudes and perceptions*: Knowledge about health effects of smoking / smokeless tobacco use.

Sample Design

The sample for GATS is selected using a multi-stage, geographically clustered design to ensure adequate coverage of the entire target population while simultaneously minimizing data collection costs. The target population for GATS includes all men and women age 15 years or older who consider the country to be their primary place of residence. The GATS design excludes the institutionalized population, tourists, and persons at a military base or group quarters from the study population. Extreme remote or unsafe areas might also be excluded from the target population, provided a significant proportion of the country's population does not reside in these areas.

The first phase of GATS is designed to produce estimates at the national level, by urban/rural classification, by gender and by the cross of gender and urban/rural with a 95% margin of error of 3 percentage points or less for tobacco use rates of 40%, and should be sufficiently large to detect differences between survey rounds with independently chosen samples. Assuming a design effect of 2.00 for estimates computed at the national level, by urban/rural classification, by gender and by the cross of gender and urban/rural, the minimum sample sizes needed to accommodate these precision requirements are 2,000 respondents in each of the four groups defined by the cross of urban/rural and gender. This results in a minimum expected respondent sample of 8,000 which is subject to applicable nonresponse and ineligibility at each stage.

Further information about GATS can be found at:

U.S. Centers for Disease Control and Prevention: www.cdc.gov/tobacco/global/gats

World Health Organization: www.who.int/tobacco/surveillance/gats/en/index.html

Appendix B: GATS Questionnaire Development Background

Concept Formation and Initial Draft of Questionnaire

Initial GATS questions were developed at the first GATS protocol development meeting in Geneva during February 2007. The development process included a review of tobacco questions from existing surveys such as the International Tobacco Control Policy Evaluation Project (the ITC Project), Adult Tobacco Survey Household Questionnaire (ATS), Stepwise Approach to Chronic Disease Risk Factor Surveillance (STEPs), and World Health Survey (WHS). A decision was made to craft the tobacco prevalence questions in a similar manner to STEPs. A draft questionnaire was produced shortly after this meeting.

Testing and Development of Questionnaire

GATS questionnaire development and cognitive pretesting was conducted in February through May 2007. This included two rounds of cognitive interviewing in order to test and revise the questionnaire. Cognitive interviewing was conducted in order to provide insights about the respondents' understanding of survey questions and terms, recall strategies, and difficulties associated with the response task, and to generate recommendations to improve the questions. RTI International conducted the cognitive interviews in Washington, DC and Research Triangle Park, NC and assisted with questionnaire development. In May 2007, the GATS paper questionnaire was piloted in two small pretests, one in India and one in the Philippines.

Consultation Workshop to Refine Questionnaire

Following the pretests in India and the Philippines, the GATS questionnaire was reviewed and revised during a partner meeting in Atlanta in June 2007. Results from cognitive testing and pretests in India and the Philippines were discussed. The GATS core questionnaire and listing of optional questions was initially finalized in October 2007.

Development of Electronic Data Collection for GATS

In November and December 2007, GATS partners decided to use electronic data collection as the mode of administration of the GATS questionnaire (instead of paper and pencil). An important reason for using electronic mode of administration was to help increase data quality by incorporating a program with features such as automated skip logic, range checks, and consistency checks. RTI International was selected as the contractor to provide programming and IT support. RTI's General Survey System (GSS) software was used to program the GATS questionnaire for handheld computers. Development and testing of the GATS electronic questionnaire occurred from January through April 2008.

In-Country Pretests and Refinements to Core Questionnaire

Further refinements were made to the GATS core questionnaire based on three meetings:

- GATS Analysis Workshop held in Atlanta in July 2008. Purpose was to discuss analysis indicators produced from GATS, which led to a few proposed questionnaire refinements.
- GATS Pretest Debriefing Workshop held in Poland in August 2008. Purpose was to debrief with countries that had completed a pretest using the GATS questionnaire.
- GATS Technical Review & Options Consultation meeting held in Atlanta in November 2009. Purpose was to review data quality issues from ongoing GATS surveys for future refinement.

Final Core Questionnaire

The final version of the GATS Core Questionnaire was locked down in August 2008. This version was fielded in the main study for all 14 initial GATS countries. (Note: The GATS Core Questionnaire was slightly updated in November 2010 for future GATS implementation.)

Country-Specific Adaptations and Optional Questions

Participating countries may add and/or adapt questions to the GATS Core Questionnaire related to the country-specific situation. This includes selecting additional questions from a list of optional questions and/or adding in new questions that the country produces. The questionnaire is translated into local country language(s) and back-translated into English for review (as needed). The in-country GATS team, consisting of people from the Ministry of Health (MoH) and implementing agency, work together with consultation from WHO and CDC technical staff to create the specific country questionnaire with adaptations. An in-country technical advisory committee and the MoH will provide final approval of the country-specific GATS questionnaire based on pretest recommendations.

GATS Questionnaire Review Committee (QRC)

The GATS QRC, a group comprised of experts in tobacco control and questionnaire design from developed and developing countries, reviews and approves all GATS questionnaires. The QRC works closely with each country to adapt the GATS questionnaire for each country's situation (e.g., identifying appropriate country-specific analysis indicators), while maintaining the standard GATS core questions to ensure comparability across countries.

Appendix C: Question Mapping to GATS

The tobacco questions in this guide are taken from the GATS Core Questionnaire. Reference to the GATS Core Questionnaire numbering is provided below.

Question Number	Reference to GATS Q#	Description
Q1	B01	Current tobacco smoking status
Q2a	B02	Past daily smoking status (for current less than daily smokers)
Q2b	B03	Past smoking status (for current non-smokers)
Q3 (a-g)	B06/B10 (a-g)	Number of tobacco products smoked per day
Q4	C01	Current smokeless tobacco use
Q5a	C02	Past daily smokeless use (for current less than daily users)
Q5b	C03	Past smokeless use (for current non-users)
Q6	E03	Frequency of anyone smoking at home
Q7	E04	Currently work outside home
Q8	E05	Work indoor/outdoor
Q9	E08	Anyone smoke at work during the past 30 days
Q10	D01	Tried to quit in past 12 months
Q11	D04	Visiting a doctor in past 12 months
Q12	D07	Receiving advice to quit smoking from doctor
Q13	G01a	Noticing anti-cigarette info in newspapers or magazines
Q14	G01b	Noticing anti-cigarette info on television
Q15	G02	Noticing health warnings on cigarette packs
Q16	G03	Thinking about quitting because of health warnings
Q17	G04a	Noticing cigarette advertisements in stores
Q18 (a-f)	G06 (a-f)	Noticing cigarette promotions
Q19	F01	Last cigarette purchase – quantity
Q20	F02	Last cigarette purchase – cost

Acknowledgements

GATS partners wish to acknowledge the contributions made by several collaborators in researching and developing the proposed set of key tobacco questions. The generous time and effort of the following is highly appreciated: Ben Apelberg (JHSPH), Samira Asma (CDC), Ron Borland (Cancer Council Victoria), Jennifer Ellis (Bloomberg Philanthropies), Daniel Ferrante (WHO), Gary Giovino (University at Buffalo, State University of New York), Prakash Gupta (Healis – Sekhsaria Institute For Public Health), Mostafa Mohammed (Egyptian Smoking Prevention Research Institute), Jeremy Morton (CDC), Krishna Mohan Palipudi (CDC).

The financial support of the Bloomberg Initiative to Reduce Tobacco Use (a program of Bloomberg Philanthropies through the CDC Foundation) is gratefully acknowledged along with the contributions made by Jeremy Morton (CDC) who wrote and organized the initial draft of the booklet.

In addition, special thanks are extended to Edouard Tursan d'Espaignet (WHO) and Lubna Bhatti (WHO) for reviewing, Oksana Bilukha (CDC) for coordination and editing, and Akiko Wilson (CDC) for preparing the layout design.

GATS Collaborating Organizations:

- U.S. Centers for Disease Control and Prevention (CDC)
- CDC Foundation
- Johns Hopkins Bloomberg School of Public Health (JHSPH)
- RTI International
- University of North Carolina Gillings School of Public Health (UNCSPH)
- World Health Organization (WHO)

Contact Information

The *Tobacco Questions for Surveys* booklet is available for download from CDC's website at: http://www.cdc.gov/tobacco/global/gats/ or WHO's website at: http://www.who.int/tobacco/surveillance/en/.

For queries on this document, please contact:

CDC

Dr. Samira Asma
Office on Smoking and Health
Centers for Disease Control and Prevention
4770 Buford Highway, N.E., MS K-39
Atlanta, GA 30341
United States of America
tobaccoinfo@cdc.gov

WHO

Dr. Douglas Bettcher
Tobacco Free Initiative
World Health Organization
Avenue Appia
Geneva 1211
Switzerland
tfi@who.int
