# Supplementary material for: Lifestyle patterns and their nutritional, socio-demographic and psychological determinants in a community-based study: A mixed approach of latent class and factor analyses
Source: PLoS One. 2020 Jul 23;15(7):e0236242. doi: 10.1371/journal.pone.0236242 (PMC7377498; doi:10.1371/journal.pone.0236242)
Supplement: S6 File — (PDF) [file pone.0236242.s007.pdf]

بررسی اثر بخشی برنامه بهبود شیوه زندگی

پرسشنامه استعمال دخانیات (Global Tobacco Surveillance System(GTSS (۱)

Q1- آیا شما در حال حاضر از سیگار، پیپ یا چپق استفاده می کنید؟

۱)بطور روزانه (Q2) ☐ ۲) برخی از روزها (Q1a) ☐ ۳)هرگز (Q1b) ☐ ٤)نمی‌دانم (Q2) ☐

Q1a- آیا شما در گذشته به طور روزانه از سیگار، پیپ یا چپق استفاده می‌کردید؟

۱)بلی (Q2) ☐ ۲) خیر(Q2) ☐ ۳)نمی دانم (Q2) ☐

Q1b–آیا شما در گذشته به طور روزانه یا برخی از روزها از سیگار، پیپ یا چپق استفاده می‌کردید؟

۱) بطور روزانه ☐ ۲) برخی از روزها ☐ ۳) هرگز ☐ ٤) نمی‌دانم ☐

Q2- آیا شما در حال حاضر از قلیان استفاده می کنید؟

۱)بطور روزانه (Q3) ☐ ۲)برخی از روزها (Q2a) ☐ ۳)هرگز (Q2b) ☐ ٤)نمی‌دانم (Q3) ☐

Q2a- آیا شما در گذشته به طور روزانه از قلیان استفاده می‌کردید؟

۱)بلی (Q3) ☐ ۲) خیر(Q3) ☐ ۳)نمی دانم (Q3) ☐

Q2b–آیا شما در گذشته به طور روزانه یا برخی از روزها از قلیان استفاده می‌کردید؟

۱)بطور روزانه ☐ ۲) برخی از روزها ☐ ۳)هرگز ☐ ٤) نمی‌دانم ☐

Q3- آیا شما در حال حاضر از سیگار ، پیپ یا چپق بدون دود استفاده می کنید؟

۱)بطور روزانه (Q4) ☐ ۲)برخی از روزها (Q3a) ☐ ۳) هرگز (Q3b) ☐ ٤) نمی‌دانم (Q4) ☐

Q3a- آیا شما در گذشته به طور روزانه از سیگار ، پیپ یا چپق بدون دود استفاده می کردید؟

۱)بلی (Q4) ☐ ۲)خیر (Q4) ☐ ۳)نمی دانم (Q4) ☐

Q3b- آیا شما در گذشته به طور روزانه یا برخی از روزها از سیگار، پیپ یا چپق بدون دود استفاده می‌کردید؟

۱)بطور روزانه ☐ ۲)برخی از روزها☐ ۳) هرگز☐ ٤) نمی‌دانم ☐

Q4- آیا شما در حال حاضر از قلیان بدون دود استفاده می کنید؟

۱)بطور روزانه (Q5) ☐ ۲) برخی از روزها (Q4a) ☐ ۳)هرگز (Q4b) ☐ ٤)نمی‌دانم (Q5) ☐

Q4a- آیا شما در گذشته به طور روزانه از قلیان بدون دود استفاده می کردید؟

۱)بلی (Q5) ☐ ۲)خیر (Q5) ☐ ۳) نمی دانم (Q5) ☐

Q4b- آیا شما در گذشته به طور روزانه یا برخی از روزها از قلیان بدون دود استفاده می‌کردید؟

۱)بطور روزانه ☐ ۲)برخی از روزها ☐ ۳)هرگز ☐ ٤)نمی‌دانم ☐

Q5- آیا کسی در خانه از سیگار، پیپ یا چپق استفاده می کند؟

۱)بله، بطور روزانه ☐ ۲) بله، چند بار در هفته ☐ ۳)بله، چند بار در ماه☐ ٤)بلی، چند بار در سال ☐ ٥)هرگز ☐ ٦)نمی دانم ☐

Q6- آیا کسی در خانه از قلیان استفاده می کند؟

۱)بله، بطور روزانه ☐ ۲)بله، چند بار در هفته ☐ ۳) بله، چند بار در ماه ☐ ٤)بلی، چند بار در سال ☐ ٥)هرگز ☐ ٦)نمی دانم☐

Q7- آیا شما در حال حاضر خارج از خانه کار می کنید؟ ۱)بلی (Q8) ☐ ۲) خیر(Q10) ☐

Q8- شما معمولا در چه مکانی کار می کنید؟ سر بسته یا سر باز ؟ ۱)سر بسته(Q9) ☐ ۲)سرباز (Q10) ☐ ۳)هر دو(Q9) ☐

Q9- آیا در یک ماه گذشته کسی در مکان سربسته ای که شما کار می کنید سیگار ، پیپ یا چپق و یا قلیان کشیده است؟

۱)بلی ☐ ۲)خیر☐ ۳)نمی‌دانم ☐

Q10- آیا در یک ماه گذشته شما در مجلات و روزنامه ها پیام هایی درباره خطرات دخانیات و در مورد تشویق به ترک دخانیات دیده اید؟

۱) بلی☐ ۲) خیر☐ ۳) روزنامه یا مجله نمی‌خوانم ☐

بررسی اثر بخشی برنامه بهبود شیوه زندگی

Q11- آیا در یک ماه گذشته شما در تلویزیون پیام هایی درباره خطرات دخانیات و یا در مورد تشویق به ترک دخانیات دیده اید؟

۱)بلی☐ ۲) خیر☐ ۳) تلویزیون تماشا نمی کنم☐

Q12- آیا در یک ماه گذشته متوجه پیامی در رابطه با خطرات استعمال دخانیات بر روی پاکت های سیگار شده اید؟

۱)بلی☐ ۲) خیر☐ ۳) پاکت سیگار ندیده ام☐

Q13- در یک ماه گذشته متوجه کدام یک از تبلیغات سیگار شده اید؟ ۱)ارائه سیگار رایگان ☐ ۲)حراج سیگار☐ ۳)ارائه جایزه و یا تخفیف سایر اجناس در صورت خرید سیگار ☐ ٤) ارائه کوپن برای سیگار ☐ ۵) لباس و یا هر چیز دیگری که مارک سیگار و یا شکل سیگار داشته باشد☐ ٦) تبلیغات سیگار در ایمیل☐ ۷)تابلوی مغازه ها☐ ۸) ویترین مغازه ها ☐

Q14- آیا فرد زیر ۱۵ سالی در خانه از سیگار، پیپ یا چپق استفاده می کند؟ ۱) بلی ☐ ۲)خیر ☐ ۳)نمی‌دانم ☐

Q15- آیا فرد زیر ۱۵ سالی در خانه از قلیان استفاده می کند؟ ۱) بلی☐ ۲)خیر ☐ ۳) نمی‌دانم☐

Q16- آیا تا به حال از مشروبات الکلی استفاده کرده اید؟ ۱) بلی (Q17)☐ ۲)خیر (Q18) ☐ ۳) نمی‌دانم (Q18) ☐

Q17- آیا شما در حال حاضر از مشروبات الکلی استفاده می کنید؟

۱)بطور روزانه ☐ ۲)برخی از روزها☐ ۳)هرگز ☐ ٤) نمی‌دانم ☐

Q18- آیا تا به حال از مواد مخدر استفاده کرده اید؟ ۱)بلی (Q19)☐ ۲)خیر (Q20)☐ ۳) نمی‌دانم (Q20)☐

Q19- شما تا به حال از کدام نوع مواد مخدر استفاده کرده اید؟

Q20- آیا شما در حال حاضر از مواد مخدر استفاده می کنید؟

۱)بطور روزانه☐ ۲)برخی از روزها ☐ ۳)هرگز ☐ ٤) نمی‌دانم ☐

-اگر فرد در حال حاضر و یا در گذشته از سیگار یا قلیان استفاده می کند سوالات زیر پرسیده شود.

Q21-آیا در یک سال گذشته اقدام به ترک داشته اید؟ ۱)بلی ☐ ۲) خیر ☐

Q22- آیا در یک سال گذشته با پزشک و یا مراقب بهداشتی ملاقات داشته اید؟ ۱) بلی(Q23)☐ ۲)خیر (Q24)☐

Q23- آیا در یک سال گذشته پزشک و یا مراقب بهداشتی شما را به ترک سیگار تشویق کرده است؟ ۱)بلی ☐ ۲)خیر☐

Q24- آیا در یک ماه گذشته دیدن برچسب های اخطار دهنده در مورد عوارض مصرف سیگار بر روی پاکت های سیگار شما را بر آن داشته است که در مورد ترک سیگار فکر کنید؟

۱)بلی☐ ۲)خیر☐ ۳)نمی‌دانم ☐

Q25- آیا شما در یک ماه گذشته تبلیغات سیگار را از جایی که سیگار می خریدید دیده اید؟

۱)بلی☐ ۲)خیر☐ ۳)از جایی سیگار نمی خرم☐

Q26- در آخرین باری که برای خود سیگار خریده اید چند سیگار خریداری کرده اید؟ ۱)تعداد سیگار .... ۲)تعداد بسته (وچند سیگار داخل بسته بوده است) .... ۳)تعداد کارتن (و چند سیگار داخل کارتن بوده است) .... ٤)سایر موارد: .... ۵)هرگز سیگار نمی خرم

Q27- آخرین باری که سیگار خریده اید چه مقدار پول بابت آن پرداخت کرده اید؟ ....

Q28- سیگار را بیشتر از کدام محل خریداری می کنید؟ ۱)سوپر مارکت ☐ ۲)دکه های روزنامه فروشی☐ ۳)فروشندگان دوره گرد☐ ٤) غیره.....

Q29- قلیان را بیشتر در کدام محل استعمال می کنید؟ ۱)خانه☐ ۲)قهوه خانه ☐ ۳) مکان های تفریحی ☐ ٤) غیره.....

Q30- بطور متوسط چه مقدار از مواد دخانی زیر را مصرف می کنید؟ ۱) سیگار تجاری(نخ): ...... بار در روز/هفته ۲) سیگار دست ساز: .... بار در روز/هفته ۳)کرتک (سیگار اندونزیایی): ....بار در روز/هفته ٤)پیپ یا چپق پر از تنباکو: .... بار در روز/هفته ۵) قلیان: .... بار در روز/هفته
