# Supplementary material for: Lifestyle patterns and their nutritional, socio-demographic and psychological determinants in a community-based study: A mixed approach of latent class and factor analyses
Source: PLoS One. 2020 Jul 23;15(7):e0236242. doi: 10.1371/journal.pone.0236242 (PMC7377498; doi:10.1371/journal.pone.0236242)
Supplement: S7 File — (PDF) [file pone.0236242.s008.pdf]

دانشگاه علوم پزشکی و خدمات بهداشتی درمانی تبریز

Tabriz University of Medical Sciences

Short form of International Physical Activity Questionnaire (IPAQ-SF)

We are interested in finding out about the kinds of physical activities that people do as part of their everyday lives. The questions will ask you about the time you spent being physically active in the **last 7 days**. Please answer each question even if you do not consider yourself to be an active person. Please think about the activities you do at work, as part of your house and yard work, to get from place to place, and in your spare time for recreation, exercise or sport.

Think about all the **vigorous** activities that you did in the **last 7 days**. **Vigorous** physical activities refer to activities that take hard physical effort and make you breathe much harder than normal. Think *only* about those physical activities that you did for at least 10 minutes at a time.

1. During the **last 7 days**, on how many days did you do **vigorous** physical activities like heavy lifting, digging, aerobics, or fast bicycling?

   _____ **days per week**

   ☐ No vigorous physical activities → ***Skip to question 3***

2. How much time did you usually spend doing **vigorous** physical activities on one of those days?

   _____ **hours per day**

   _____ **minutes per day**

   ☐ Don't know/Not sure

Think about all the **moderate** activities that you did in the **last 7 days**. **Moderate** activities refer to activities that take moderate physical effort and make you breathe somewhat harder than normal. Think only about those physical activities that you did for at least 10 minutes at a time.

3. During the **last 7 days**, on how many days did you do **moderate** physical activities like carrying light loads, bicycling at a regular pace, or doubles tennis? Do not include walking.

   _____ **days per week**

   ☐ No moderate physical activities → ***Skip to question 5***

4. How much time did you usually spend doing **moderate** physical activities on one of those days?

_____ **hours per day**

_____ **minutes per day**

☐ Don't know/Not sure

Think about the time you spent **walking** in the **last 7 days.** This includes at work and at home, walking to travel from place to place, and any other walking that you have done solely for recreation, sport, exercise, or leisure.

5. During the **last 7 days**, on how many days did you **walk** for at least 10 minutes at a time?

_____ **days per week**

☐ No walking → ***Skip to question 7***

6. How much time did you usually spend **walking** on one of those days?

_____ **hours per day**

_____ **minutes per day**

☐ Don't know/Not sure

The last question is about the time you spent **sitting** on weekdays during the **last 7 days**. Include time spent at work, at home, while doing course work and during leisure time. This may include time spent sitting at a desk, visiting friends, reading, or sitting or lying down to watch television.

7. During the **last 7 days**, how much time did you spend **sitting** on a **week day**?

_____ **hours per day**

_____ **minutes per day**

☐ Don't know/Not sure

This is the end of the questionnaire, thank you for participating.
