# Supplementary material for: Lifestyle patterns and their nutritional, socio-demographic and psychological determinants in a community-based study: A mixed approach of latent class and factor analyses
Source: PLoS One. 2020 Jul 23;15(7):e0236242. doi: 10.1371/journal.pone.0236242 (PMC7377498; doi:10.1371/journal.pone.0236242)
Supplement: S8 File — (PDF) [file pone.0236242.s009.pdf]

پرسشنامه بین المللی فعالیت بدنی (٤)

Q1- در طول ۷ **روز اخیر** چند روز آن فعالیت بدنی **شدید** مانند بلند کردن اجسام سنگین، حفاری (مثل کندن باغچه)، ایروبیک (ورزش هوازی)، دوچرخه‌سواری سریع، فوتبال و دویدن داشته‌اید؟

- ۱) .... روز در هفته ۲)فعالیت بدنی شدید نداشته‌ام ☐ (مراجعه به سؤال Q3)

Q2- معمولاً چه مدت زمانی را در چنین روزهایی برای انجام این فعالیت‌های بدنی **شدید** به صورت پیوسته صرف کرده‌اید؟

- ۱)............ ساعت در روز ۲) .... دقیقه در روز

فعالیت‌های بدنی **متوسطی** را که در طول ۷ **روز اخیر** انجام داده‌اید، مدنظر قرار دهید. فعالیت‌های فیزیکی **متوسط** به فعالیت‌هایی اطلاق می‌شود که قدرت متوسطی می‌خواهد و باعث می‌شود شما کمی تندتر از حالت عادی نفس بکشید.

لطفاً فقط فعالیت‌هایی را مدنظر قرار دهید که **حداقل به مدت ۱۰ دقیقه به صورت پیوسته** انجام داده‌اید.

Q3- در طول ۷ **روز اخیر** چند روز آن فعالیت فیزیکی **متوسط** مانند حمل بارهای سبک، دوچرخه‌سواری با سرعت متوسط یا والیبال انجام داده‌اید؟ **لطفاً پیاده‌روی را به حساب نیاورید.**

- ۱)............ روز در هفته ۲) فعالیت بدنی متوسط نداشته‌ام ☐ (مراجعه به سؤال Q5)

Q4- معمولاً چه مدت زمانی را در چنین روزهایی برای انجام فعالیت‌های بدنی **متوسط** صرف کرده‌اید؟

- ۱)............ ساعت در روز ۲)............. دقیقه در روز

لطفاً مدت زمانی را که در طول ۷ **روزگذشته** به **پیاده‌روی** اختصاص داده‌اید، مدنظر قرار دهید. این قسمت پیاده روی در محل کار، در خانه، برای رفتن از محلی به محل دیگر و هر نوع پیاده روی دیگر که شما به عنوان تفریح، ورزش، تمرینات جسمانی یا در اوقات فراغت انجام داده‌اید را شامل می‌شود.

Q5- در طول ۷ **روز اخیر**، چند روز آن به **مدت حداقل به مدت ۱۰ دقیقه و به صورت پیوسته پیاده‌روی** داشته‌اید؟

- ۱)............ روز در هفته ۲)پیاده‌روی نداشته‌ام ☐ (مراجعه به سؤال Q7)

Q6- معمولاً چه مدت زمانی را در چنین روزهایی برای **پیاده‌روی** صرف کرده اید؟

- ۱)............ ساعت در روز ۲)............. دقیقه در روز ۳)نمی‌دانم/ مطمئن نیستم

آخرین سؤال مربوط به اوقاتی است که شما در طول ۷ **روز اخیر** به **نشستن** اختصاص داده‌اید که شامل نشستن در محل کار، در خانه، هنگام انجام تکالیف و در اوقات فراغت می‌باشد. این زمان نشستن پشت میز، نشستن یا لم دادن هنگام تماشای تلوزیون و مطالعه و زمانی که برای نشستن با دوستان و فامیل اختصاص داده‌اید را هم شامل می‌شود.

Q7- در طول ۷ **روز اخیر**، چه مدت زمانی را در **هر روز** به **نشستن** اختصاص داده‌اید؟

۱) .... ساعت در روز ۲) .... دقیقه در روز ۳) نمی‌دانم/ مطمئن نیستم
