# Supplementary material for: Lifestyle patterns and their nutritional, socio-demographic and psychological determinants in a community-based study: A mixed approach of latent class and factor analyses
Source: PLoS One. 2020 Jul 23;15(7):e0236242. doi: 10.1371/journal.pone.0236242 (PMC7377498; doi:10.1371/journal.pone.0236242)
Supplement: Attachment — Submitted filename: Response to Reviewers.docx [file pone.0236242.s010.docx]

**Ms. Ref. No.:  PONE-D-19-32691	20 March, 2020**

**Title: Lifestyle patterns and their nutritional, socio-demographic and psychological determinants in a community-based study: A mixed approach of latent class and factor analyses**

Dear Professor Paul Gerard Shiels

I hope you are well. Thank you very much for your close attention to our submitted manuscript. I have revised the manuscript according to the reviewers’ comments. Also, following is our response to the reviewer’s comments. The comments by reviewers are presented in black color and the responses from the authors are presented in blue color. The comments which were given alongside the papers of the manuscript have been corrected and marked with yellow color.

Yours sincerely,

Mahdieh Abbasalizad Farhangi, Ph.D.

Associate Professor

Drug Applied Research Center,

Tabriz University of Medical Sciences,

Tabriz, Iran

**Journal Requirements**:

When submitting your revision, we need you to address these additional requirements.

1. Please ensure that your manuscript meets PLOSONE's style requirements, including those for file naming. The PLOS ONE style templates can be found at <http://www.journals.plos.org/plosone/s/file?id=wjVg/PLOSOne_formatting_sample_main_body.pdf> and <http://www.journals.plos.org/plosone/s/file?id=ba62/POSOne_formatting_sample_title_authors_affiliations.pdf>

Action or explanation: Thank you for your comment. The correction has been made as requested.

2. We suggest you thoroughly copyedit your manuscript for language usage, spelling, and grammar. If you do not know anyone who can help you do this, you may wish to consider employing a professional scientific editing service.

Action or explanation: Thank you for your comment. The correction has been made as requested.

Whilst you may use any professional scientific editing service of your choice, PLOS has partnered with both American Journal Experts (AJE) and Editage to provide discounted services to PLOS authors. Both organizations have experience helping authors meet PLOS guidelines and can provide language editing, translation, manuscript formatting, and figure formatting to ensure your manuscript meets our submission guidelines. To take advantage of our partnership with AJE, visit the AJE website (<http://learn.aje.com/plos/>) for a 15% discount off AJE services. To take advantage of our partnership with Editage, visit the Editage website ([www.editage.com](http://www.editage.com)) and enter referral code PLOSEDIT for a 15% discount off Editage services.  If the PLOS editorial team finds any language issues in text that either AJE or Editage has edited, the service provider will re-edit the text for free.

Upon resubmission, please provide the following:

• The name of the colleague or the details of the professional service that edited your manuscript
• A copy of your manuscript showing your changes by either highlighting them or using track changes (uploaded as a *supporting information* file)

• A clean copy of the edited manuscript (uploaded as the new *manuscript* file)

Action or explanation: Thank you for your comment. The correction has been made as requested.

3. Please include additional information regarding the survey or questionnaire used in the study and ensure that you have provided sufficient details that others could replicate the analyses. For instance, if you developed a questionnaire as part of this study and it is not under a copyright more restrictive than CC-BY, please include a copy, in both the original language and English, as Supporting Information.

Action or explanation: Thank you for your comment. The correction has been made as requested.

4. Thank you for stating the following on the title page of your manuscript:

"The work has been granted by Research Undersecretary of Tabriz University of Medical Sciences (Registration number: IR.TBZMED.REC.1398.077)."

We note that you have provided funding information that is not currently declared in your Funding Statement. However, funding information should not appear in the Acknowledgments section or other areas of your manuscript. We will only publish funding information present in the Funding Statement section of the online submission form.

Please remove any funding-related text from the manuscript and let us know how you would like to update your Funding Statement. Currently, your Funding Statement reads as follows:
 "The funders had no role in study design, data collection and analysis, decision to publish, or preparation of the manuscript."

Action or explanation: Thank you for your comment. The correction has been made as requested.

5 Please include your tables as part of your main manuscript and remove the individual files. Please note that supplementary tables (should remain/ be uploaded) as separate "supporting information" files

Action or explanation: Thank you for your comment. The correction has been made as requested.

6. In your Data Availability statement, you have not specified where the minimal data set underlying the results described in your manuscript can be found. PLOS defines a study's minimal data set as the underlying data used to reach the conclusions drawn in the manuscript and any additional data required to replicate the reported study findings in their entirety. All PLOS journals require that the minimal data set be made fully available. For more information about our data policy, please see <http://journals.plos.org/plosone/s/data-availability>.

Upon re-submitting your revised manuscript, please upload your study’s minimal underlying data set as either Supporting Information files or to a stable, public repository and include the relevant URLs, DOIs, or accession numbers within your revised cover letter. For a list of acceptable repositories, please see <http://journals.plos.org/plosone/s/data-availability#loc-recommended-repositories>. Any potentially identifying patient information must be fully anonymized.

Important: If there are ethical or legal restrictions to sharing your data publicly, please explain these restrictions in detail. Please see our guidelines for more information on what we consider unacceptable restrictions to publicly sharing data: <http://journals.plos.org/plosone/s/data-availability#loc-unacceptable-data-access-restrictions>. Note that it is not acceptable for the authors to be the sole namedindividuals responsible for ensuring data access. We will update your Data Availability statement to reflect the information you provide in your cover letter.

Action or explanation: Thank you for your comment. The correction has been made as requested.

[Note: HTML markup is below. Please do not edit.]

Reviewers' comments:

Reviewer's Responses to Question

**Comments to the Author**

1. Is the manuscript technically sound, and do the data support the conclusions?

The manuscript must describe a technically sound piece of scientific research with data that supports the conclusions. Experiments must have been conducted rigorously, with appropriate controls, replication, and sample sizes. The conclusions must be drawn appropriately based on the data presented.

Reviewer#1: Partly

Reviewer #2: No

2. Has the statistical analysis been performed appropriately and rigorously?
Reviewer#1: Yes
Reviewer #2: No

3. Have the authors made all data underlying the findings in their manuscript fully available?
The [PLOS Data policy](http://www.plosone.org/static/policies.action#sharing) requires authors to make all data underlying the findings described in their manuscript fully available without restriction, with rare exception (please refer to the Data Availability Statement in the manuscript PDF file). The data should be provided as part of the manuscript or its supporting information, or deposited to a public repository. For example, in addition to summary statistics, the data points behind means, medians and variance measures should be available. If there are restrictions on publicly sharing data—e.g. participant privacy or use of data from a third party—those must be specified.

Reviewer#1: Yes

Reviewer #2: No

4. Is the manuscript presented in an intelligible fashion and written in standard English?

PLOS ONE does not copyedit accepted manuscripts, so the language in submitted articles must be clear, correct, and unambiguous. Any typographical or grammatical errors should be corrected at revision, so please note any specific errors here.

Reviewer #1: Yes

Reviewer #2: No

5. Review Comments to the Author

Please use the space provided to explain your answers to the questions above. You may also include additional comments for the author, including concerns about dual publication, research ethics, or publication ethics. (Please upload your review as an attachment if it exceeds 20,000 characters)
Reviewer #1:

This paper is well written and uses a novel data set based on 525 adults from East Azarbaijan, Iran. The main issue I have with the paper is that it is highly unusual to utilize a mental health variable such as anxiety as 'lifestyle behavior'. Lifestyles behaviors are normally smoking, dietary intake, physical activity and alcohol. Anxiety is often associated with lifestyle behaviors such as smoking, diet and physical activity but is not normally included as a 'lifestyle behavior' itself. I think the authors need to revise their research questions and re-analyse their data to exclude anxiety (although they may wish to explore the association between anxiety and smoking, diet and physical activity).

Action or explanation: Thank you for your comment. You are absolutely right. Anxiety status was excluded from the latent class analysis. Please see the revised manuscript.

Reviewer #2:

The authors state that the data underlying the study is fully available without restriction; however the following question looking for details of where it is available has not been completed correctly.
Action or explanation: Thank you for your comment. The correction has been made as requested.

The source of funding statement needs re-phrased.

Action or explanation: Thank you for your comment. The correction has been made as requested.

There is a massive contradiction in the introduction, the author's state that other studies focus on single lifestyle factors in isolation then continue to cite several articles that look at these in combination or show correlations between them.

Action or explanation: Thank you for your comment. We corrected it in the text. Please see the revised manuscript.

A copy of the questions/questionnaire should be presented with the manuscript so that the validity of the questions can be assessed by the reader.

Action or explanation: Thank you for your comment. The correction has been made as requested.

The authors state that individuals with physical disability, severe illness and a few other conditions were excluded but have not provided figures for these exclusions, where they excluded from the larger study and thus were not present in the cohort or where they excluded precisely from this study?

Action or explanation: Thank you for your comment. Subjects with physical disability, severe chronic illness requiring bed rest, mental disability, active liver injury, and pregnant women were not included in our study.

The restriction to Iranian nationals limits the results and conclusions to this narrow focus - invalidating most of the global seeming claims in the abstract, this is hinted at in the conclusions but should be clearly and concisely stated.

Action or explanation: Thank you for your comment. The paper abstract was corrected. Please see the revised manuscript.

It is stated that 2 blood pressure measurements were taken and then averaged, the standard deviations should also be stated to ensure these were not skewed by other factors e.g. stress.

Action or explanation: Thank you for your comment. The standard deviation of blood pressure is listed in Table 3.

No information about collection of blood samples has been provided i.e. was there anti-coagulant added to the blood or was the sample clotted naturally? Were any other additives added to the blood? Catalogue numbers should be included.

Action or explanation: Thank you for your comment. The correction has been made as requested. Please see the revised manuscript .After a 12–14 h overnight fasting, 10 ml venous blood samples were collected from all participants and placed in vacutainer/ siliconized test tubes without anticoagulant for biochemical determinations.

Alanine amino transferase is ALT, not AST and no definition is provided for AST. The ultraviolet measurement method is not described or referenced.

Action or explanation: The corrections had been made as requested. Please see the revised manuscript.

"Binary indicators (healthy versus unhealthy behavior) were created based on existing recommendations and/or propositions by the literature." Needs to be properly referenced with examples.

Action or explanation: Thank you for your comment. The corrections had been made as requested. Please see the revised manuscript.

Anxiety status, defined in methods as mild, moderate and severe; but referred to elsewhere as low, moderate and high - consistency in description.

Action or explanation: Thank you for your comment. The manuscript had been revised accordingly. Please see the revised manuscript. Scores of 0-5, 6-9, 10-14 and ≥15 specify normal, low, moderate and high symptoms of anxiety, respectively.

Under smokers there is no provision for passive smoking, former smokers or heavy/light smokers.
Action or explanation: Thank you for your comment. Tobacco consumption was defined as daily smoking of at least one cigarette, cigar, pipe or cigarillo within the last 30 days and a binary indicator for smoking was created (never smoked versus current and former smokers). Never smoked and current and former smokers are listed in Table 3.

Descriptive statistics - MANOVA should be used for multiple testing not one-way ANOVA and there is no correction for multiple testing.

Action or explanation:

Factor analysis- 21 food groups are mentioned, then 23? Need to be accurate and consistent. Part of this section repeats itself.

Action or explanation: Thank you for your comment. We sincerely apologize for the mistake. The manuscript had been revised accordingly. Please see the revised manuscript.

Latent Class Analysis - anxiety status is listed as a dichotomous factor, but under description there are 3 categories defined - clarification needed as to what this represents. The article relies very heavily on latent class analysis (LCA), however a detailed description of the process and references are required to support its use and implementation. Adjustment for multiple comparisons is needed, if any were performed these have not been described. "related to the signs" needs to be clearly defined. This whole section is lacking in references and description.

Action or explanation: The manuscript had been revised accordingly. Please see the revised manuscript. Thank you for your comment. Anxiety status was excluded from the latent class analysis. Scores of 0-5, 6-9, 10-14 and ≥15 specify normal, low, moderate and high symptoms of anxiety, respectively. The LCA is used to identify the latent classes and unknown patterns based on a set of observed variables from multivariate classified data.

Theuse of "Western dietary pattern" is inaccurate and not an accepted description while this may be an accepted local colloquialism perhaps it would be more accurate, descriptive and appropriate to refer to "unhealthy diet pattern".

Action or explanation: Thank you for your comment. The manuscript had been revised accordingly. Please see the revised manuscript

The three groupings or classes are severely unbalanced casting doubt on the statistical significance of these and on the study design.

Action or explanation: Thank you for your comment

All 3 groups had low probabilities of smoking and anxiety disorder - if these were particularly low then they should have been excluded as factors in the analysis, or the analysis should focus on proportionate levels between the groups and the whole cohort. Given the "low" status of these factors in all groups it can't be that these were used to define the categories or that they had any impact at all.
Action or explanation: Thank you for your comment. You are right. Anxiety status was excluded from the latent class analysis.

Traditional dietary pattern: how organ and red meat was defined as this can be categorized as unprocessed and process red meat e.g., pate will be both red meat and organ meat, but it is processed. No list of processed meat was included in table 1. It is generally accepted that processed vs unprocessed meats are a risk/discriminant factor when examining diet, this has not been mentioned let alone addressed in this manuscript.

Action or explanation: Thank you for your comment. For food pattern determination, a quantitative food frequency questionnaire (FFQ) was used. The FFQ included 80 questions which was developed and validated previously and reference portions were defined based on the most reported portion sizes in the 24-h recall. Based on the validation of the questionnaire, the people in this region use very little processed meat. Therefore processed meats are not included in our FFQ.

Ref.

Nikniaz L, Tabrizi J, Sadeghi-Bazargani H, Farahbakhsh M, Tahmasebi S, Noroozi S. Reliability and relative validity of short-food frequency questionnaire. British Food Journal. 2017.

Definition of the 150 clusters is required.

Action or explanation: Thank you for your comment. The manuscript had been revised accordingly. Please see the revised manuscript. This study conducted by probability proportional to size (PPS) multistage stratified cluster sampling. Clusters comprise one to several blocks or parts of blocks. Blocks were usually attached buildings.

More detailed definition of metabolic equivalents (MET -minutes/week) is required, Anxiety status needs expansion and more detailed description.

Action or explanation: Thank you for your comment. The manuscript had been revised accordingly. Please see the revised manuscript.

It would be nice to include some visual representation of data normality e.g., the frequency distribution (histogram), stem-and-leaf plot, boxplot, P-P plot (probability-probability plot), or Q-Q plot (quantile-quantile plot). Similarly a visual representation of the overall analysis would be beneficial as would the actual PCA plots from the PCA analysis.

Action or explanation: Thank you for your comment.

No description of meaning of factor analysis with varimax rotation and why PCA, more basic exploratory analysis was used?

Action or explanation: Thank you for your comment. The manuscript had been revised accordingly. Please see the revised manuscript. Distinguishing between overlapping components is a main reason why PCA has been used. The varimax rotation was used because it has the potential to minimize the number of components. The PCA is a method for finding a lower-dimensional representation, which accounts for the majority of the variance in the features. Factor analysis with varimax rotation is performed to develop a more interpretable solution. The rotated solution uses a PCA of reduced dimensionality as the starting point. In general, rotation needed because it makes the factor structures meaningful and easily interpretable.

Both methods allow reducing the dimensionality of datasets; however EEA is designed with the objective to identify certain unobservable factors from the observed variables, whereas PCA at best, PCA provides an approximation to the required factors.

How was the factor score calculated?

Action or explanation: Thank you for your comment. Please see the revised manuscript. The factor score for each pattern was calculated by summing consumption of all food groups weighted by their factor loadings.

"Most of them had moderate physical activity (45%)" this is less than half, it is inaccurate to state "most"
Action or explanation: Thank you for your comment. We sincerely apologize for the mistake. Please see the revised manuscript.

The article in general massively over interprets the results and makes large claims that are unsupported by the data, the large spread of factors across all groups indicates that these are unlikely to be significant factors in their own right within this cohort and therefore only very specific combinations may be valid but these were not explored at all.

Action or explanation: Thank you for your comment.

The supposed healthy group actually had the highest levels of smoking and anxiety, comparisons between groups and to the whole cohort would be very telling in this instance.

Action or explanation: Thanks for the comment. You are absolutely right. In our study, participants in the first class have a healthy dietary pattern and moderate physical activity, but we can't say that they have a perfectly healthy lifestyle.

Was gender investigated as a discriminatory factor?

Action or explanation: Thank you for your comment

The conclusions are far fetched and unsupported, especially given the severe limitations of the study.

 Action or explanation: Thank you for your comment. The manuscript had been revised accordingly. Please see the revised manuscript

6. PLOS authors have the option to publish the peer review history of their article ([what does this mean?](https://journals.plos.org/plosone/s/editorial-and-peer-review-process#loc-peer-review-history)). If published, this will include your full peer review and any attached files.
If you choose “no”, your identity will remain anonymous but your review may still be made public.
**Do you want your identity to be public for this peer review?** For information about this choice, including consent withdrawal, please see our [Privacy Policy](https://www.plos.org/privacy-policy).

Reviewer#1: No

Reviewer#2: No

[NOTE: If reviewer comments were submitted as an attachment file, they will be attached to this email and accessible via the submission site. Please log into your account, locate the manuscript record, and check for the action link "View Attachments". If this link does not appear, there are no attachment files to be viewed.]
